# Bridging the accuracy-speed divide in reactive molecular dynamics with QuantaMind MD

**DOI:** 10.1126/sciadv.aeg3595

**Published:** 2026-09-11

**Authors:** Song Xia, Deqiang Zhang, Xu Shang, Jinbo Xu

**Affiliations:** MoleculeMind, Beijing, China.

## Abstract

Simulating chemical reactivity remains a central challenge in molecular dynamics, historically constrained by the trade-off between ab initio accuracy and classical efficiency. We present QuantaMind, a machine learning force field (MLFF) framework that attains density functional theory (DFT)–level accuracy, enabling fully reactive simulations of complex molecular systems. QuantaMind achieves exceptional numerical stability over tens-of-nanosecond trajectories, maintaining accuracy throughout long-time reactive dynamics. It reproduces spontaneous bond formation and cleavage in key chemical processes—including proton transfer, acid-base neutralization, and phosphate buffering—and captures biologically essential phenomena such as histidine titration under constant pH. As a demonstration of the framework’s applicability to enzyme catalysis, QuantaMind recapitulates the complete catalytic cycle of the PETase-catalyzed hydrolysis reaction. By uniting quantum-level accuracy with computational efficiency comparable to state-of-the-art MLFFs, QuantaMind establishes a paradigm for long-timescale, fully reactive molecular simulation, opening avenues for rational catalyst design, reactive materials discovery, and predictive modeling of biochemical function.

## INTRODUCTION

Molecular dynamics (MD) simulations are indispensable tools in computational biomolecular modeling, providing atomistic insights into the structure, dynamics, and function of complex molecular systems (*1*, *2*). In a typical MD simulation, atomic trajectories are computed by evaluating interatomic forces at each time step and integrating Newton’s equations of motion. Classical force fields (FFs) use empirical functional forms to approximate system energies and forces. Although they enable simulations of large biomolecular systems, their accuracy is limited by fixed parameters and simplifications that fail to account for electronic effects, particularly in reactive environments.

The most accurate approach to calculating interatomic forces involves solving the electronic Schrödinger equation (SE). However, analytical solutions exist only for systems with very few degrees of freedom, and high-accuracy numerical solutions via quantum mechanics (QM) are computationally prohibitive for large-scale simulations (*3*). Machine learning force fields (MLFFs) bypass the need to solve the electronic SE directly by learning the mapping from molecular geometries to energies and forces using supervised learning approaches (*4*). This offers several orders of magnitude in speedup relative to QM methods. Recent MLFFs have shown considerable promise in both chemical and biomolecular domains (*5*–*9*). However, many are trained on equilibrium or near-equilibrium conformations sampled from classical FF simulations. While a growing number of MLFFs incorporate nonequilibrium and reactive configurations, many are focused on small-molecule reactions involving only a few heavy atoms and do not readily extend to large biomolecular systems. Consequently, their accuracy often degrades when applied to out-of-distribution systems, such as those involving chemical reactions or diverse elemental compositions.

Building MLFFs for reactive systems is still an active area of research. For example, Transition1x (*10*) made a substantial step forward by incorporating 9.6 million density functional theory (DFT) calculations across 10,000 organic reaction pathways. Building on this, several reactive MLFFs have been developed that target transition state (TS) optimization in small molecules (*11*, *12*).

In this study, we introduce QuantaMind, a deep learning–based MLFF framework designed for fully reactive MD simulations in biomolecular systems. Trained on a curated set of TS-centered configurations at nonequilibrium geometries, QuantaMind captures key reaction dynamics in both pure water and the PETase enzyme. Furthermore, following established active learning practices in MLFF development (*13*–*15*), simulation-generated trajectories, including those from biased simulations, are used to iteratively augment the training set. With targeted training on phosphate ions and related species, we show that QuantaMind successfully reproduces biologically essential phenomena, such as histidine titration under constant pH. Overall, this work establishes QuantaMind as a general-purpose, AI-driven platform for reactive atomistic modeling, combining DFT-level accuracy with computational efficiency comparable to state-of-the-art MLFFs.

## RESULTS

### The QuantaMind framework

#### Training data generation and model training

Figure S1 summarizes the development and application cycle of the QuantaMind framework. The process begins with biomolecular simulations of the system of interest, which serve to generate diverse conformational ensembles for subsequent DFT calculations. Classical MD simulations are commonly used at this stage to explore large biomolecular systems. TS optimization using high-level QM methods is additionally used to access configurations involving bond breaking and formation. Both approaches are used in this work to generate relevant molecular geometries (for details, see the “TS dataset construction” and “HIS, Na^+^, PBS, and PETase dataset construction” sections).

Following initial conformational sampling, the next step involves preparing systems for QM calculations. This is particularly important for large structures generated during classical or QuantaMind MD simulations, where computational cost is substantial. QM properties—including energies, forces, and dipole moments—are calculated using DFT methods. These properties, paired with input geometries, constitute the training data for QuantaMind, a deep learning–based MLFF optimized for energy and force prediction.

**Fig. 1.**
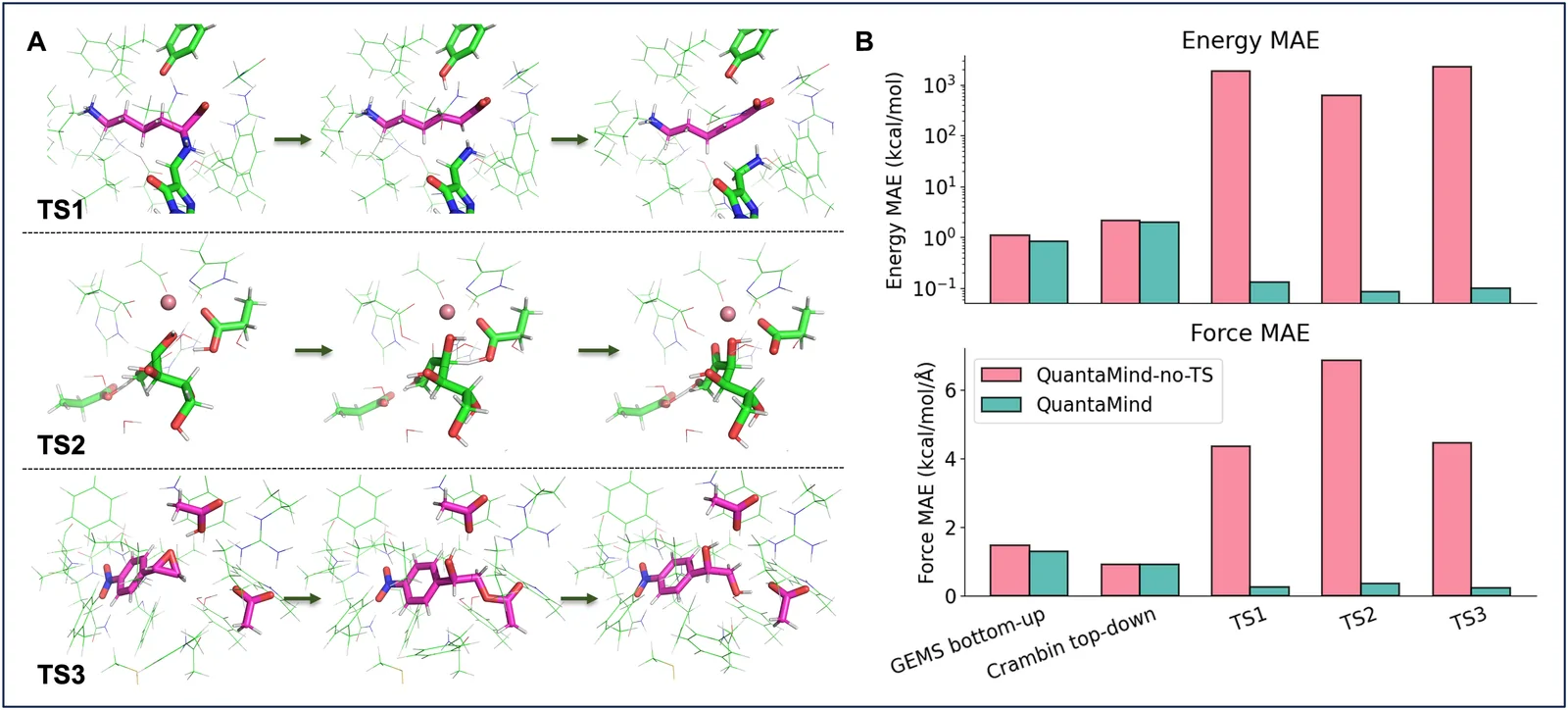
QuantaMind achieves state-of-the-art performance on protein fragments and TS datasets. (**A**) Overview of datasets developed in this work. TS1, TS2, and TS3 are TS datasets described in the “TS dataset construction” section. (**B**) Model performance on two public datasets and the three TS datasets. GEMS bottom-up and Crambin top-down were introduced in the GEMS work (*8*).

The first iteration of QuantaMind is trained on a combination of three TS datasets (Fig. 1A) developed herein, along with the GEMS bottom-up and Crambin top-down protein fragment datasets (see table S1). Figure 1B illustrates the model’s performance before and after training on these datasets. The results show that incorporation of TS-specific data improves accuracy on corresponding test sets, highlighting the importance of reaction-centric data for simulating reactive systems. Notably, the TS datasets were originally developed for independent studies and were not curated specifically for this project; they were selected on the basis of their inclusion of relevant chemical elements at nonequilibrium geometries.

#### Comparison with existing MLFFs

Because most existing MLFFs are benchmarked on unrelated datasets, direct comparisons are difficult. To enable a fair comparison, we trained a separate QuantaMind model using only the SO3LR training data, excluding all other datasets developed in this work, and evaluated its performance against SO3LR (*16*). As shown in fig. S2, QuantaMind and SO3LR perform comparably across all four benchmark test sets; the differences are minor throughout. We attribute these differences to distinct model architectures, random weight initialization, and training hyperparameters. Besides, we do not train QuantaMind on Hirshfeld ratio labels for convenience. As highlighted by Wang *et al.* (*17*), the utility of MLFFs is no longer bottlenecked by prediction accuracy; accordingly, we do not aim to compare QuantaMind with existing MLFFs in terms of accuracy but, instead, focus on its application to MD simulations of complex biomolecular systems.

#### QuantaMind MD simulation

Once trained, the QuantaMind model is used to perform MD simulations. At each time step, atomic forces are obtained from the model’s predicted potential energy surface (PES) and used to integrate Newton’s equations of motion, advancing the system forward in time. This process generates a simulation trajectory describing the full atomic-level evolution of the system. Crucially, because QuantaMind is trained on DFT-level data that explicitly covers nonequilibrium and TS geometries, bond-breaking and formation events arise naturally during the simulation. In terms of computational cost, QuantaMind achieves an inference time of 4.2 × 10^−6^ s per atom per step on an NVIDIA A100 80GB GPU, comparable to state-of-the-art MLFFs (fig. S3).

#### Iterative training scheme

The QuantaMind framework supports an iterative training loop. The MD trajectories themselves may serve as sources of additional training data. Additional training configurations are extracted from QuantaMind simulation trajectories using the same fragmentation algorithm described in the “HIS, Na^+^, PBS, and PETase dataset construction” section. To demonstrate this, we extracted conformations from the PETase acylation step and generated 226 additional training configurations through system-specific fragmentation and DFT calculations. As shown in fig. S4, model performance on the PETase test split improves considerably after the inclusion of this data, achieving lower mean absolute error while maintaining strong generalization on other test sets. This iterative training method allows QuantaMind to be continually refined and adapted to previously unidentified biomolecular systems. In our internal testing, we observed that the model underperforms on molecules with distinct chemistries not represented in the training data. In such cases, the iterative update strategy is particularly effective for restoring predictive accuracy. This iterative training scheme resembles the active learning workflows used in recent reactive MLFF development, such as DEAL (*13*), ArcaNN (*14*), and Asparagus (*15*), which standardize the exploration of high-energy regions of the PES.

### Ab initio simulation of proton diffusion, acid-base neutralization, and water dissociation

#### Proton diffusion simulation

Proton transfer plays a critical role in many biological and chemical systems. One of the most notable properties of protons is their unusually high diffusion coefficient in water. The Grotthuss mechanism (*18*), which involves successive proton hopping between adjacent water molecules, is now widely accepted as the underlying process for proton mobility. Accurate determination of the proton diffusion coefficient via ab initio simulations remains challenging due to the long simulation times required for statistical convergence (*19*) and finite-size effects inherent to MD simulations (*20*). Although previous efforts have used MLFFs to model proton diffusion, these typically yield results in only qualitative agreement with experimental values (*21*).

**Fig. 2.**
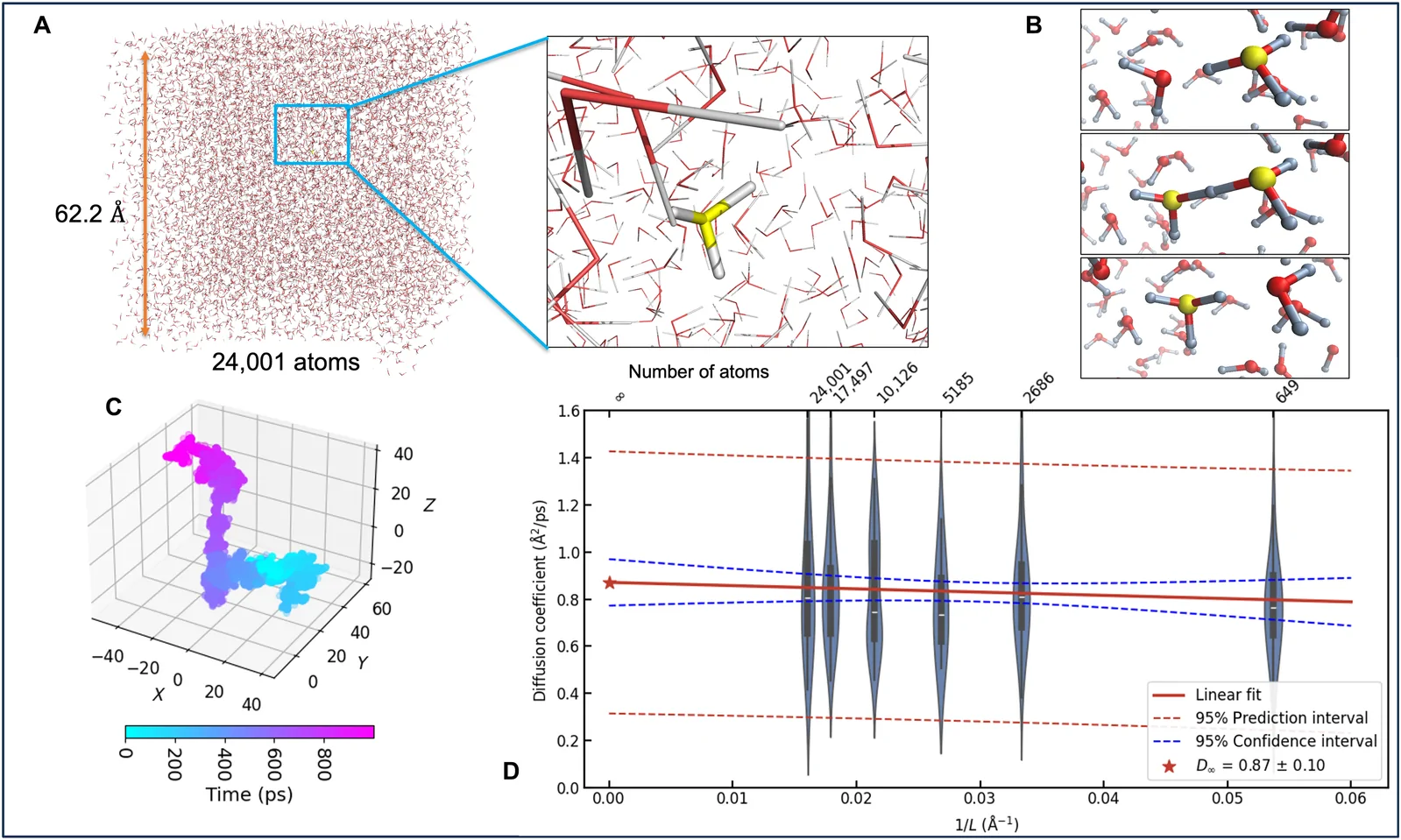
Simulation of proton diffusion in water using QuantaMind. (**A**) Visualization of the largest water box (24,001 atoms), with the hydronium ion highlighted in yellow. (**B**) Example of a proton jumping event showing transient formation of a Zundel ion. (**C**) Proton trajectory from a 1-ns simulation in the 649-atom water box, with color indicating simulation time. (**D**) Proton diffusion coefficients estimated via bootstrapping, shown as violin plots against inverse box length 1/*L* (see Materials and Methods for details). The red solid line shows the linear extrapolation to 1/*L* = 0; the red star marks the infinite-size diffusion coefficient $D_{\infty }$. Red dashed lines indicate the 95% prediction interval; dashed blue lines indicate the 95% confidence interval (CI), which is used to report the final estimate.

Figure 2 summarizes our simulations of proton diffusion. Simulations were conducted using six different box sizes, ranging from 649 to 24,001 atoms, to account for finite-size effects (*20*). For each box size, three independent 1-ns simulations were performed. The largest simulation box contains 24,001 atoms, where one water molecule is manually converted into a hydronium ion by the addition of a single proton (Fig. 2A). More than 10,000 proton transfer events are observed in each 1-ns trajectory. One such event is illustrated in Fig. 2B, where the proton is transiently shared between two water molecules, forming a Zundel intermediate. An animated simulation of proton hopping in a 649-atom water box is provided in the Supplementary Materials.

The trajectory of the hydronium ion is tracked by following the oxygen atom covalently bonded to three hydrogen atoms. Figure 2C visualizes the proton’s unwrapped trajectory over a 1-ns simulation, with color indicating simulation time. Figure 2D presents the estimated diffusion coefficients plotted against the inverse box lengths. A linear extrapolation is applied to obtain the diffusion coefficient in the infinite-system limit (*20*). From this extrapolation, we estimate a diffusion coefficient of 0.87 ± 0.10 Å^2^/ps [95% confidence interval (CI)], which is in close agreement with experimental values of 0.96 and 0.94 Å^2^/ps (*22*, *23*). For comparison, a recent study by Huo *et al.* (*21*) trained an MLFF for simulating proton transfer in pure water and reported a diffusion coefficient of 0.375 ± 0.242 Å^2^/ps. Previous simulation studies by Paesani and co-workers using multistate empirical valence bond models reported diffusion coefficients in the range of 0.29 to 0.50 Å^2^/ps (*24*, *25*). AIMD simulations using various DFT functionals report proton diffusion coefficients of 0.28 to 0.33 Å^2^/ps (PW91, BLYP, and HCTH/120), 1.08 ± 0.27 Å^2^/ps (PBE), 1.28 ± 0.19 Å^2^/ps (PBE-TS), and 0.83 ± 0.19 Å^2^/ps (PBE0-TS) (*26*). Notably, the QuantaMind model used in our simulation was not explicitly trained on hydronium ions; improved accuracy is likely achievable through the iterative training scheme described in the “The QuantaMind framework” section.

As pointed out by Muñoz-Santiburcio (*19*), a core difficulty in modeling proton diffusion lies in the limited statistics available per trajectory: Only a single excess proton is present in each simulation box, while the remaining particles are neutral water molecules, so a single trajectory yields only one estimate of $D_{H_{3}O^{+}}$. We also note that the bootstrapping error bars on the diffusion coefficient are likely underestimates of the true statistical uncertainty (*27*). Generating more statistically independent samples through longer or additional simulations would be required to properly converge the diffusion coefficient estimation.

#### Water autoionization simulation

The autoionization of water is a fundamental reaction in which a water molecule donates a proton to another, forming hydronium (H_3_O^+^) and hydroxide (OH^−^) ions. The dissociation constant of water, *K*_w_, is defined as

$$K_{w}=\left[H_{3}O^{+}\right]\left[{\text{OH}}^{-}\right]$$ (1)

Here, [H_3_O^+^] and [OH^−^] represent the molar concentrations of hydronium and hydroxide ions, respectively. At room temperature, $K_{w}=1.0\times 10^{-14}$, corresponding to concentrations of 1.0 × 10^−7^ M for both ions and a neutral pH of 7. At 373 K (100°C), *K*_w_ increases, and the pH of pure water decreases accordingly to 6.13 ± 0.03 (*28*).

**Fig. 3.**
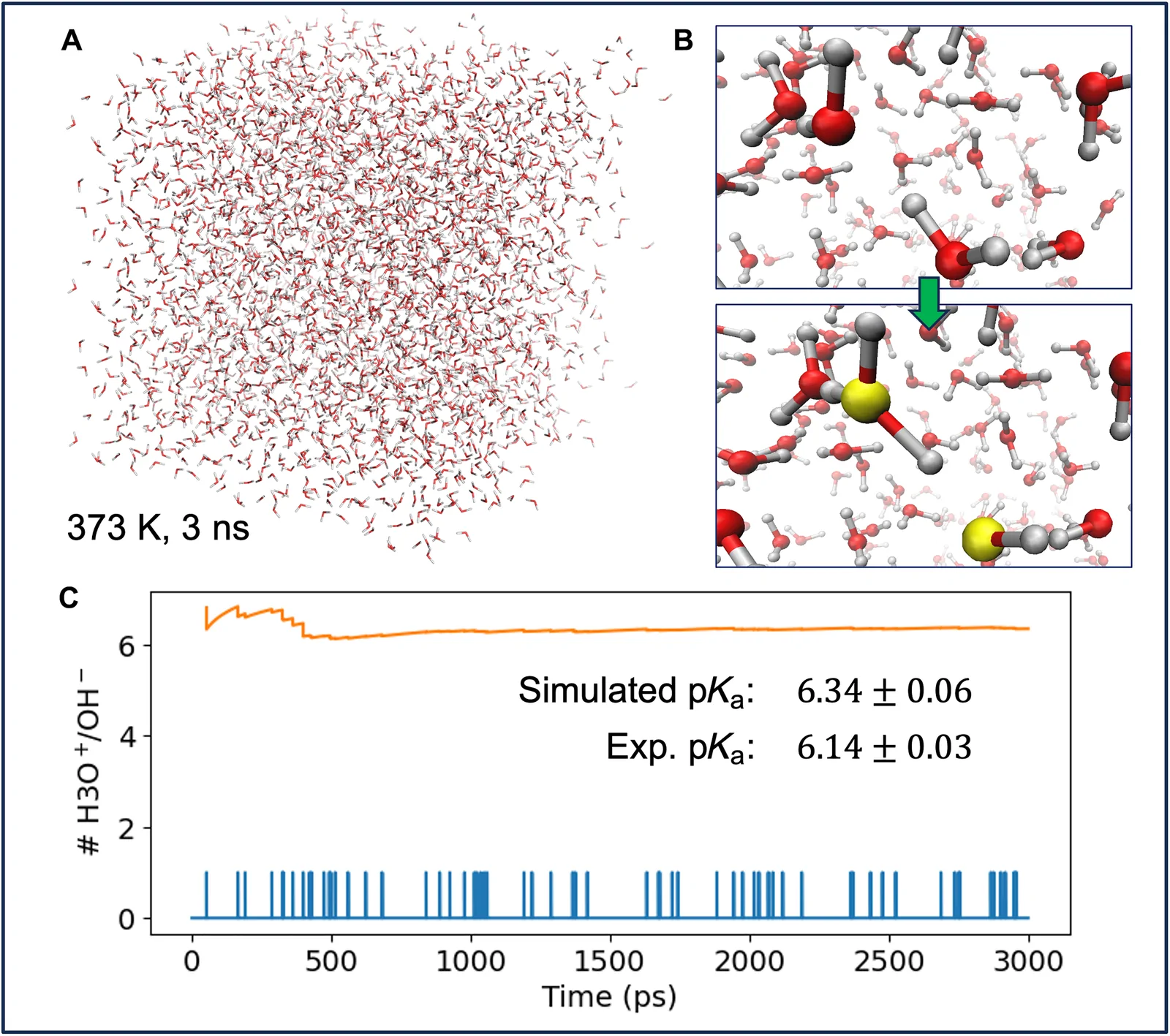
Sampling of water autoionization events at 373 K using QuantaMind. (**A**) Simulation box containing 9999 atoms (3333 water molecules). (**B**) Visualization of an autoionization event during the simulation. (**C**) Time series showing the number of H_3_O^+^ and OH^−^ ions (blue) and calculated pH (yellow) over a 3-ns trajectory.

Figure 3 summarizes our simulation of spontaneous water autoionization events using QuantaMind MD at 373 K. The system consists of 3333 water molecules (9999 atoms). Because these dissociation events are extremely rare under ambient conditions, the simulation was conducted at an elevated temperature to increase their frequency. As shown in Fig. 3C, several ionization events were captured during the 3-ns trajectory, as indicated by a nonzero ion count (blue curve). The corresponding pH trajectory (yellow curve) was derived from cumulative ion counts normalized by system size and simulation time (see the “MD simulations with QuantaMind” section for details). The estimated average pH was 6.34 ± 0.06, in good agreement with the experimentally reported value of 6.13 ± 0.03 for pure water at 373 K (*28*). These results support the ability of QuantaMind to accurately model rare yet foundational processes in water chemistry. Because pH depends logarithmically on the hydronium concentration, the pH estimate is only weakly sensitive to the number of observed autoionization events. Statistically converging the predicted pH to high precision is, therefore, inherently difficult on accessible simulation timescales.

#### Neutralization simulation

Acid-base neutralization is the reaction in which hydronium and hydroxide ions react to form water. Unlike typical diffusion-limited processes, acid-base neutralization proceeds at extraordinarily fast rates (*29*), enabled by rapid ion transport along the hydrogen-bond network and electrostatic attraction between the oppositely charged species. Because these reactions occur on picosecond timescales (*30*) and are difficult to resolve experimentally, QuantaMind simulations offer valuable insight by capturing these events atomistically and enabling direct estimation of the reaction rate constant.

As illustrated in fig. S5A, the simulation box is initialized with hydronium and hydroxide ions placed ∼10 Å apart. A total of 100 independent simulations were performed with randomized initial velocities, each lasting 50 ps. A representative neutralization event from one of the runs is shown in fig. S5B. The snapshots depict a proton transfer from the hydronium ion to a nearby water molecule, followed immediately by recombination with the hydroxide ion to form two water molecules.

Across the 100 simulation trajectories, reaction times were recorded. Figure S5C shows the resulting histogram (blue bars) and the fitted survival function based on survival analysis (*31*), a statistical method commonly used to model time-to-event data. From this model, the estimated reaction rate constant falls within the range of 4.9 × 10^11^ to 7.1 × 10^11^ M^−1^ s^−1^ (95% CI). While conventional diffusion-controlled reactions typically proceed at rates between 10^9^ and 10^10^ M^−1^ s^−1^ (*29*), our results confirm that acid-base neutralization occurs at considerably faster rates, consistent with theoretical predictions for this uniquely fast process. Hassanali *et al.* (*30*) studied the neutralization mechanism via AIMD simulations in a smaller simulation box (64 water molecules); while no rate constant was estimated, they reported characteristic recombination timescales of ∼0.5 ps. For reference, Eigen and de Maeyer (*32*) reported an experimental rate constant of 1.4 × 10^11^ M^−1^ s^−1^, which is on the same order of magnitude as our simulated estimate. We attribute this overestimation to several likely sources of error: All 100 simulations share the same initial spatial arrangement of the hydronium and hydroxide ions, so the variability in reaction times arises solely from randomized initial velocities rather than from a diverse sampling of initial configurations, introducing a systematic bias in the estimated rate constant. Besides, the relatively small simulation box and number of replicas may increase the variation of the simulation results. Addressing these limitations in a future work may further improve the simulation accuracy.

### Cinematic simulation of PETase catalytic cycle with full quantum accuracy

Polyethylene terephthalate (PET) is the most widely used thermoplastic polymer in the polyester family, commonly found in textiles, food packaging, and beverage containers. Due to its widespread use, PET accumulates in large quantities in the environment, presenting a substantial ecological concern. In 2016, a PET-assimilating bacterium, *Ideonella sakaiensis* 201-F6, was isolated from a recycling facility. This bacterium secretes a cutinase-like enzyme, IsPETase, capable of degrading PET into mono(2-hydroxyethyl) terephthalic acid (MHET), terephthalic acid (TPA), and ethylene glycol under ambient conditions (*33*). Since this discovery, substantial efforts have been devoted to improving PETase through rational design, directed evolution, and machine learning, resulting in a variety of optimized variants (*34*, *35*).

The catalytic mechanism of serine hydrolases, including PETase, has been extensively studied. However, key mechanistic steps, particularly those involving proton transfer, remain debated (*36*, *37*). While hybrid QM/molecular mechanics (QM/MM) simulations have provided valuable insights, they are also known to introduce artifacts due to limitations in the MM component and boundary effects. Moreover, QM/MM studies often require large QM regions to achieve convergence, resulting in high computational costs (*38*).

**Fig. 4.**
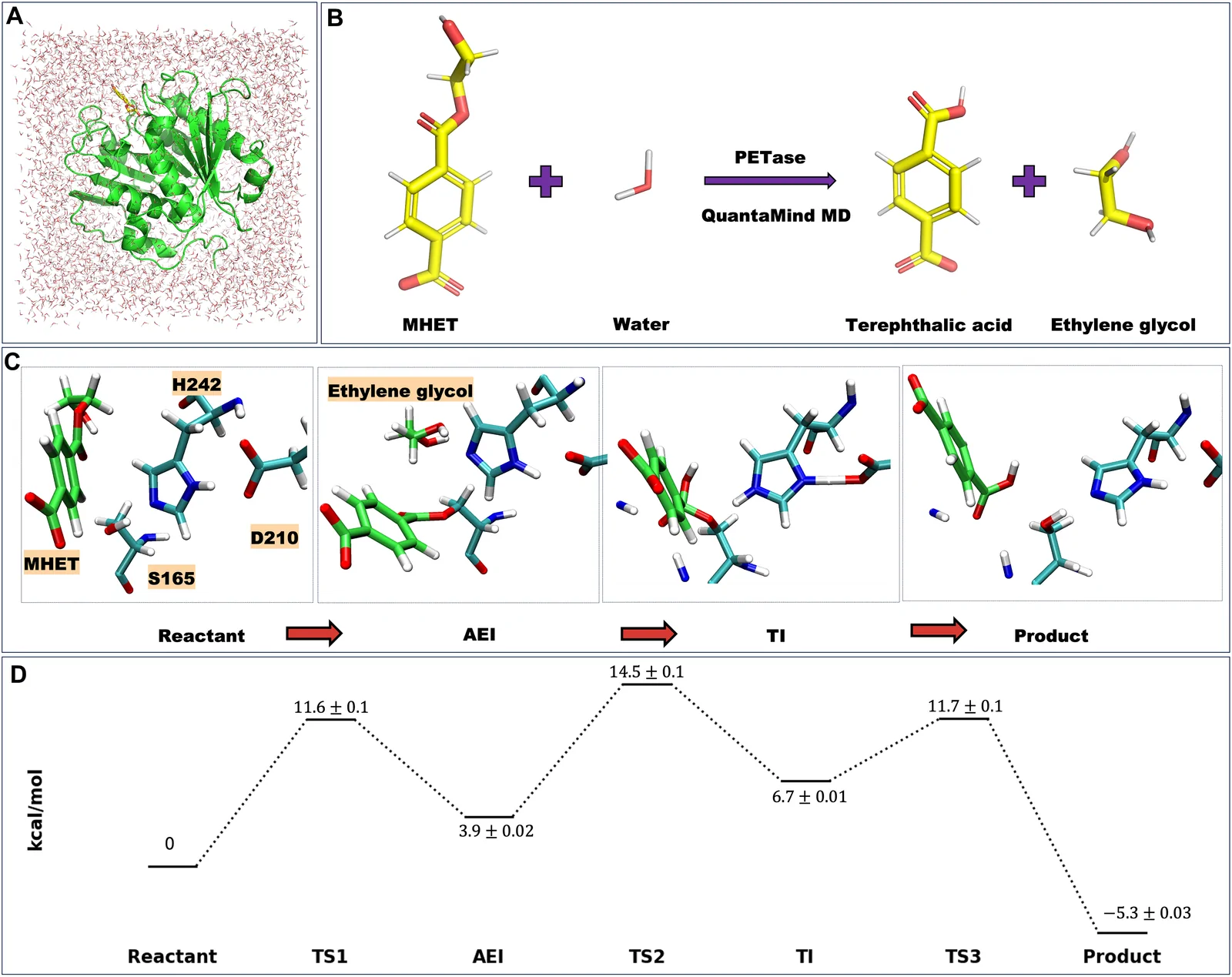
Overview of the PETase catalytic cycle simulated by QuantaMind MD. (**A**) The simulation box used in this work. (**B**) The overall chemical reaction. The three-dimensional geometries of the molecules are obtained from the simulation trajectories. (**C**) Representative frames from the simulation, indicating key intermediates and products. AEI stands for acyl-enzyme intermediate, and TI stands for tetrahedral intermediate. (**D**) Reaction barriers estimated from the simulation.

In this work, we demonstrate the application of QuantaMind to simulate the complete catalytic cycle of PETase with full QM accuracy. Figure 4 provides an overview of the enzymatic reaction modeled in this study. The simulation system (Fig. 4A) consists of 17,792 atoms, including the PETase protein (257 residues), one MHET substrate molecule, and 4898 water molecules. All atoms in the system, including solvent water molecules, are treated explicitly by the QuantaMind MLFF. Figure 4B illustrates the overall chemical reaction: hydrolysis of MHET into TPA and ethylene glycol. Figure 4C depicts representative frames extracted from the simulation trajectory, capturing key reaction intermediates observed along the catalytic pathway. Detailed analyses of each catalytic step, including energy barriers and intermediate stability, are presented in the next subsections. To explore each reaction step, umbrella sampling is used to drive the system along a chosen reaction coordinate while QuantaMind provides the underlying PES. The specific atom pairs constrained, the target distances, and the simulation durations for each step are described in detail in the “PETase catalytic cycle simulation” section.

#### PETase acylation reaction

The acylation step of the PETase catalytic cycle begins with a nucleophilic attack by the serine residue S165 on the ester bond of the MHET substrate. This initiates cleavage of the ester linkage and leads to the formation of two products: ethylene glycol and the acyl-enzyme intermediate (AEI).

Figure S6A shows representative snapshots from the nucleophilic attack simulation. Several key atomic distances were monitored to characterize the reaction coordinates (first 1800 ps of fig. S6C). In particular, two hydrogen bonds, labeled H-bond M166 and H-bond Y95, are formed between the carbonyl oxygen of MHET and the backbone amide nitrogens of M166 and Y95, respectively. These bonds represent the canonical “oxyanion hole” that stabilizes the tetrahedral intermediate of serine hydrolases. Early in the trajectory, with S165 positioned distally from MHET, the H-bond distances fluctuate considerably. As the nucleophile approaches, both hydrogen bond lengths stabilize around 3 Å, indicating enhanced structural stabilization of the intermediate.

A second key event is the proton transfer from the hydroxyl group of S165 to the $N_{\epsilon }$ atom of H242 (monitored as H242 NE–S165 H). Before proton transfer, this distance remains near 2 Å, a characteristic of a noncovalent hydrogen bond. Around 1600 ps, a sharp decrease in the distance to ∼1 Å signifies proton capture by H242, consistent with covalent N─H bond formation.

Following intermediate formation, the reaction proceeds with cleavage of the ester bond and generation of the AEI and ethylene glycol. Figure S6B shows snapshots from this stage, while key interatomic distances are tracked in the last 200 ps of fig. S6C.

The hydrogen bonds H-bond M166 and H-bond Y95 continue to act as stabilizing elements of the oxyanion hole, interacting with the evolving carbonyl group of the AEI. A central feature of this step is the proton transfer from H242 to the oxygen atom of the departing ethylene glycol. As the ester bond breaks, the glycol oxygen, initially undercoordinated, extracts a proton from H242. This event is charted in two distance plots: Glycol O–H242 HNE (oxygen-proton distance) and H242 NE–H242 HNE (nitrogen-proton distance). Around 1900 ps, a tight O─H distance (∼1 Å) and simultaneous elongation of the N─H bond (∼1.8 Å) indicate successful proton transfer, forming a stable O─H group in the glycol and regenerating the neutral histidine. A complete animation of this simulation is available in the Supplementary Materials.

For enhanced sampling, only the MHET ester C─O bond is constrained with umbrella sampling (see the “PETase catalytic cycle simulation” section) during the first 1800 ps of the simulation. No constraint is applied during the last 200 ps of the simulation. The potential of mean force (PMF), reconstructed using the weighted histogram analysis method (WHAM) (*27*), is shown in fig. S6D. The free energy barrier for this step is estimated to be 11.6 ± 0.1 kcal/mol, where the uncertainty is obtained via bootstrapping.

#### PETase deacylation reaction

In the deacylation step of the PETase catalytic cycle, a water molecule performs a nucleophilic attack on the ester bond of the AEI. This is followed by cleavage of the bond between the carbonyl carbon and the oxygen of S165, releasing TPA and restoring the original state of the catalytic triad. This reaction proceeds through two mechanistic stages, illustrated in figs. S7 and S8.

##### Stage 1: Nucleophilic attack by water

Figure S7A presents representative snapshots from the 2000-ps simulation of the nucleophilic attack by the water molecule on the AEI carbonyl carbon. Key atomic interactions were monitored over time and are shown in fig. S7B. The hydrogen bond distances labeled H-bond M166 and H-bond Y95 correspond to interactions between the AEI carbonyl oxygen and the backbone amide nitrogens of M166 and Y95, forming the classical oxyanion hole that stabilizes the negatively charged intermediate. Compared to the nucleophilic attack observed in the acylation step (fig. S6C), these hydrogen bond distances exhibit larger fluctuations, suggesting a more transiently stabilized intermediate.

A notable event during this stage is the proton transfer from the attacking water molecule to the $N_{\epsilon }$ atom of H242, occurring around 1500 ps. This results in the protonation of H242 and the activation of the water oxygen for nucleophilic attack. Throughout this stage, only the distance between the nucleophilic water oxygen and the AEI carbonyl carbon (MHET C–Water O) was constrained using umbrella sampling (see the “PETase catalytic cycle simulation” section); no additional bias was introduced. The PMF reconstructed via WHAM analysis (*27*) is shown in fig. S7C. The estimated free energy barrier for this step is 10.5 kcal/mol.

##### Stage 2: Ester bond cleavage and product release

In the second stage, cleavage of the ester bond between the AEI and S165 completes product formation and enzymatic regeneration. Figure S8A shows snapshots of this transformation. Time-resolved interatomic distances are plotted in fig. S8B. As the reaction proceeds, increased fluctuations in H-bond M166 and H-bond Y95 are observed, indicating that the product, TPA, forms weaker interactions with the oxyanion hole than the tetrahedral intermediate. A key event in this stage is the proton transfer from H242 back to S165, restoring the hydroxyl group of the catalytic serine and completing the regeneration of the active site.

Umbrella sampling was applied along the MHET C–S165 O distance, with no other constraints (see the “PETase catalytic cycle simulation” section). Figure S8C shows the corresponding PMF reconstruction from WHAM. The calculated free energy barrier for ester bond cleavage and triad restoration is 5.0 kcal/mol.

#### PETase simulation discussion

In this study, QuantaMind MD was used to simulate the complete catalytic cycle of the PETase enzyme, spanning 6 ns of atomic-scale dynamics. The simulations successfully captured key proton transfer events throughout the reaction cycle, consistent with mechanisms proposed in previous QM/MM studies (*39*, *40*). Distance analyses further underscore the critical role of the backbone amide nitrogens of Y95 and M166 in forming the oxyanion hole, which stabilizes the carbonyl oxygen atom during intermediate formation and TSs. To validate QuantaMind for these simulations, geometries were randomly sampled from each stage of the catalytic cycle—151 from the acylation step, 54 from deacylation stage 1, and 137 from deacylation stage 2—and evaluated with single-point PBE0/def2-TZVPP+MBD calculations. Parity plots (fig. S9) show Pearson correlation coefficients exceeding 0.99 for force components across all three trajectory segments, confirming that QuantaMind faithfully reproduces the PBE0/def2-TZVPP+MBD PES on configurations visited during the simulations.

Free energy profiling provided quantitative insight into the reaction energetics. Among all catalytic steps, the nucleophilic attack during the acylation phase was identified as rate limiting, with a calculated free energy barrier of 14.5 ± 0.1 kcal/mol. Experimental turnover rates for the leaf-branch compost cutinase (LCC) are mostly measured against BHET or pNP (*33*, *40*, *41*) (14.5 to 16.5 kcal/mol for pNP and 18.7 kcal/mol for BHET), while MHET is selected for simulation in this work because of the availability of structural data. We note that both nucleophilic attack and ester bond cleavage steps, during acylation and deacylation, may proceed in a concerted rather than stepwise manner (*39*). Therefore, construction of a full two-dimensional PES using multicoordinate scanning would provide a more accurate depiction of the minimum energy path. We also note that the bootstrapping error bars on the free energy barriers are likely underestimates of the true statistical uncertainty (*27*). Generating more statistically independent samples through longer or additional simulations would improve the accuracy of the error estimates but is now beyond our computational resources; we identify this as a direction for future work.

### Histidine titration at constant pH with phosphate buffering

Classical MD simulations typically account for pH indirectly by assigning fixed protonation states to titratable residues before simulation. Over the past decade, several approaches have been developed to incorporate pH effects directly, including discrete protonation state transitions (*42*, *43*) and continuous schemes such as λ-dynamics (*44*). Because QuantaMind MD can directly simulate proton transfer reactions, we evaluated its ability to model histidine titration at constant pH. Specifically, we applied QuantaMind to simulate hen egg white lysozyme (HEWL) in phosphate-buffered saline (PBS), without imposing constraints or artificial protonation states. We monitored the protonation state transitions of histidine-15 (His^15^) directly from atomic-level trajectories.

**Fig. 5.**
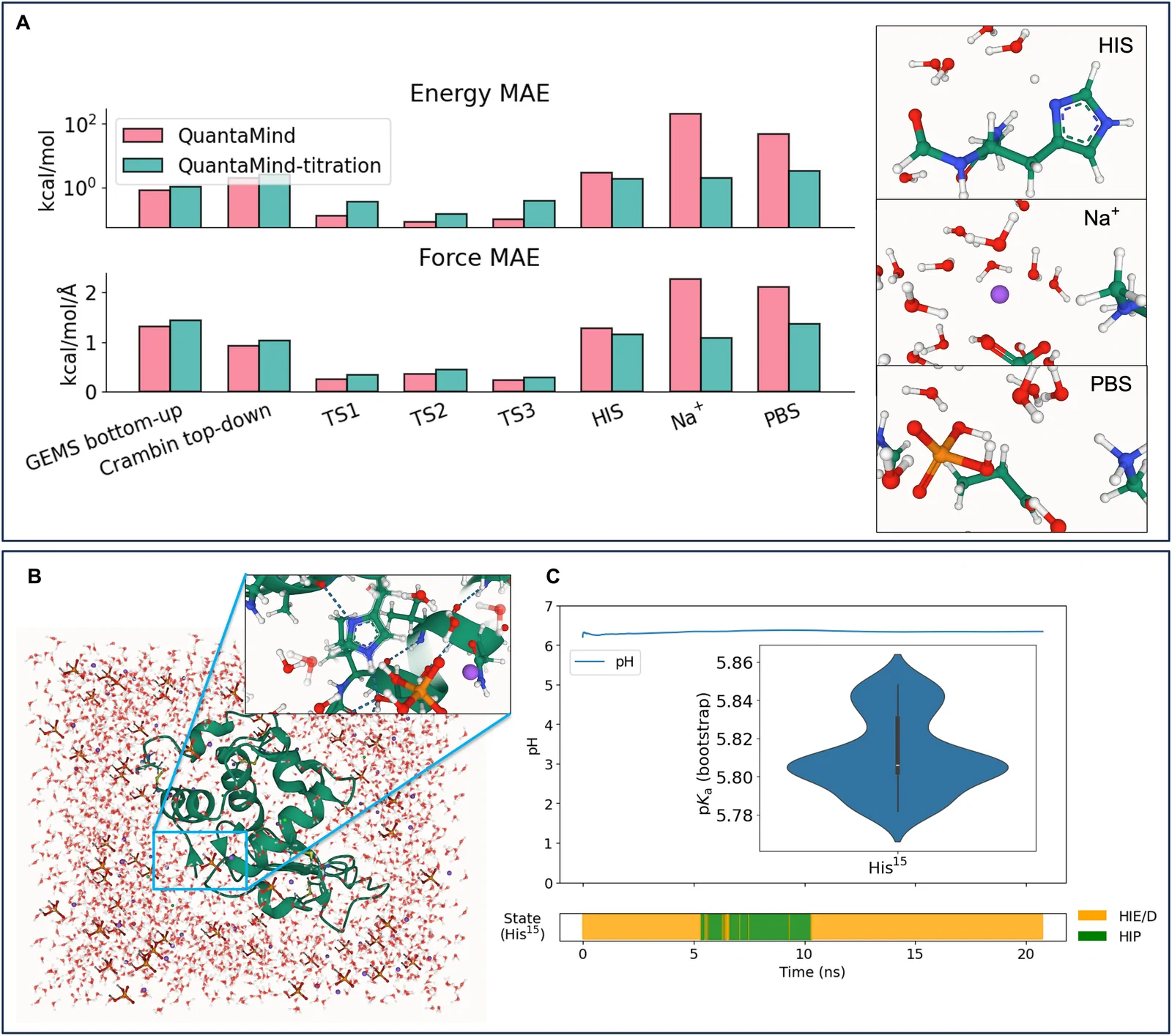
Histidine titration at constant pH simulated with QuantaMind. (**A**) Performance of the QuantaMind model after adding three training datasets prepared for histidine titration: HIS, Na^+^, and PBS. Molecular structures adjacent to the bar plots represent example fragments from each dataset. (**B**) HEWL protein in a solvated environment containing 60 Na_2_HPO_4_ and 6 NaH_2_PO_4_ molecules. The focus is on histidine-15 (His^15^). (**C**) Time series of system pH and His^15^ protonation state over the 20-ns simulation. The blue curve shows the system pH estimated via the Henderson-Hasselbalch (HH) equation. The inset shows a violin plot of p*K*_a_ values obtained by bootstrap resampling of the trajectory, illustrating the distribution of the fitted p*K*_a_ estimate. The horizontal bar at the bottom [“State (His^15^)”] is a protonation barcode: Orange indicates that His^15^ is in a neutral tautomer (HIE or HID), and green indicates the protonated (HIP) state.

Figure 5A summarizes the additional datasets prepared for the titration simulations. These include 1780 hydrogen-tunneling configurations with nonequilibrium N─H distances from simulations of histidine (HIS), 3698 fragments for Na^+^, and 1833 PBS buffer fragments collected from classical MD simulations in unrelated projects (for details, see the “HIS, Na^+^, PBS, and PETase dataset construction” section). The bar plots show that the QuantaMind model achieves improved accuracy after training on these supplemental datasets (designated as “QuantaMind-titration”) while retaining strong performance on original benchmarks. All simulations in this section are performed using this tuned model.

Figure S10A displays the simulation setup for the PBS buffering environment. Five systems with varying initial buffer compositions were simulated for 10 ns each. Ion population fluctuations were monitored during each simulation, and the corresponding species variation is shown in fig. S11, indicating ongoing ionization, hydrolysis, and buffering equilibria among the phosphate species.

To estimate the pH of the systems, we used two approaches: (i) direct counting of free hydronium ions and (ii) application of the Henderson-Hasselbalch (HH) equation using known p*K*_a_ values (where *K*_a_ is the acid dissociation constant) for phosphoric acid

$$\text{pH}=pK_{a}+\text{log}\left(\frac{\left[A^{-}\right]}{\left[\text{HA}\right]}\right)$$ (2)

Here, [A^−^] and [HA] are the molar concentrations of the conjugate base and weak acid, respectively. As shown in fig. S10B, the two pH estimates show a monotonic relationship, but visible deviations from the *y* = *x* line are present. The hydronium concentration pH oscillates substantially throughout the simulation rather than converging toward the HH-equation value. This behavior is most pronounced for higher-pH systems, where hydronium ions are rare and their instantaneous count is subject to large relative fluctuations. For example, in the Na_2_HPO_4_-dominant system, the hydronium concentration pH starts near 12.5 at 4 ns, drops to ∼9 at 7 ns (closest to the HH-equation estimate), and then drifts further away. The same oscillatory pattern is observed across all five systems. Due to the large variance of the hydronium concentration pH, the HH method is selected for subsequent pH estimations as it provides a more stable measure of the buffer pH.

Figure 5B shows the HEWL simulation system embedded in PBS buffer. The system contains 60 Na_2_HPO_4_ and 6 NaH_2_PO_4_ molecules randomly distributed around the protein. A total of 20 ns of simulation were performed, during which the protonation state of His^15^ was monitored. Using pH estimates from the buffer simulation and observed protonation population ratios, the p*K*_a_ of His^15^ was calculated via

$$pK_{a}=\text{pH}-\text{log}\left(\frac{\left[\text{HIE}\;\text{or}\;\text{HID}\right]}{\left[\text{HIP}\right]}\right)$$ (3)

Here, HIP denotes the doubly protonated (charged) state of histidine; HIE and HID represent the singly protonated (neutral) tautomers. The p*K*_a_ value was determined from cumulative statistics using bootstrap resampling of the trajectory frames.

As shown in Fig. 5C, the estimated p*K*_a_ of His^15^ is ∼5.81. This value is in good agreement with the experimentally reported p*K*_a_ of 5.5 as measured by nuclear magnetic resonance (*45*). By comparison, constant pH MD studies using conventional FFs estimate p*K*_a_ values of 4.84 ± 0.05 and 5.42 ± 0.05, depending on the FF used (*46*). These results further support QuantaMind’s ability to realistically simulate protonation equilibria and solvent-dependent titration behavior at constant pH, enabling first-principles p*K*_a_ prediction without empirical constraints. We note that protonation state transitions of His^15^ are observed predominantly between 5 and 10 ns of the trajectory, leading to an uneven sampling of protonated and neutral states. Longer simulations or the application of enhanced sampling techniques would improve the sampling of protonation state transitions and yield more statistically robust p*K*_a_ estimates.

## DISCUSSION

In this work, we introduced QuantaMind, a reactive MLFF framework that achieves DFT-level accuracy at the computational scale of classical MD. QuantaMind MD simulations successfully reproduce spontaneous bond formation and cleavage in fundamental chemical processes—including proton transfer, acid-base neutralization, water autoionization, enzymatic hydrolysis, and phosphate buffering—while capturing biologically relevant phenomena such as histidine titration under constant pH conditions.

Wang *et al.* (*17*) recently highlighted numerical stability and generalizability as persistent challenges in the deployment of MLFFs in practical settings. In this context, QuantaMind demonstrates exceptional numerical stability, sustaining tens-of-nanosecond-scale MD simulations with minimal system crashes. Errors and instabilities only become prominent when using under-trained variants of the model. To further characterize stability under conservative dynamics, we performed 1-ns NVE simulations on two pure-water systems: SPC216 (216 water molecules) and Water3333 (3333 water molecules). The total, potential, and kinetic energies, as well as the instantaneous temperature, are shown in fig. S17. The total energy drift is +7.35 × 10^−5^ kcal/mol per atom per picosecond for SPC216 and +6.74 × 10^−5^ kcal/mol per atom per picosecond for Water3333. These drift values should be interpreted with caution when comparing across models as ML-predicted energy is inconsistent between MLFFs (*47*). To the best of our knowledge, a systematic benchmark of MLFFs under NVE dynamics is not yet available; one such example is provided by Fu *et al.* (*47*), which reports an energy conservation test over 50 fs. Overall, QuantaMind achieves energy conservation over the nanosecond timescale, making it suitable for large-scale, long-time production simulations in biochemical applications.

One current challenge of QuantaMind is its relatively narrow training domain, which is largely based on protein data. Expanding chemical coverage remains an active area of investigation (*48*, *49*), and several comprehensive QM datasets have been released in recent years (*12*, *50*). Incorporating these datasets into the QuantaMind training pipeline could improve the model’s transferability across broader chemical and biological domains, particularly in applications such as protein-ligand simulations.

For TS modeling, curated benchmark datasets now provide broad chemical and conformational diversity (*10*, *12*). The development of MLFFs tailored for TS optimization remains an emerging research area (*11*, *12*), although current efforts are predominantly focused on small organic molecules. Although QuantaMind is primarily designed for reactive MD simulations in biomolecular systems, integrating such TS datasets may enhance its robustness and extend its capabilities to related tasks, such as reaction pathway mapping or transition barrier estimation.

On system-specific tasks, QuantaMind supports an effective iterative training scheme. In this approach, the general QuantaMind model is first used to generate simulation trajectories. These trajectories are then processed through system-specific fragmentation and DFT calculations to produce additional, high-quality training data. The refined model is retuned for that system, improving predictive performance. This iterative refinement is applicable not only at the chemical level—e.g., for new molecules—but also for accurately modeling diverse TS conformations obtained through enhanced sampling. As such, this strategy supports transferability to a wide range of biomolecular systems, including those for which little or no training data are initially available. Combining the QuantaMind framework with active learning workflows such as DEAL (*13*) and ArcaNN (*14*) represents a natural direction for future development.

An important advantage of the QuantaMind framework lies in its independence from force-field parameterization and QM region selection, both of which are critical and often delicate components of conventional QM/MM simulations. The model requires no manual specification of a quantum region, streamlining simulation preparation and ensuring robustness during production runs. In addition, because MLFFs such as QuantaMind deliver substantial computational speedup over traditional QM methods (*9*), greater configurational sampling and denser umbrella sampling windows can be used, improving the convergence and resolution of free energy landscapes. Together, these features demonstrate QuantaMind’s capability to simulate full enzymatic reaction pathways at DFT-level accuracy while mitigating artifacts associated with modeling boundaries or parameter tuning, enabling practical, high-fidelity enzyme modeling across broad chemical environments.

Looking forward, we envision that this MLFF-based methodology will not only complement but ultimately supersede traditional QM/MM approaches, eliminating the need for artificial system partitioning while retaining quantum-level accuracy at accessible computational cost. With continued refinement, QuantaMind MD has the potential to establish a paradigm for studying enzymatic catalysis and biomolecular reactivity.

## MATERIALS AND METHODS

### QuantaMind model architecture

Over the past decade, various MLFF architectures have been developed, ranging from descriptor-based models (*51*) to message-passing neural networks (*7*, *52*). Recently, models have aimed to incorporate explicit electronic degrees of freedom (*53*) in addition to nuclear degrees of freedom considered in previous models. Building upon the SpookyNet architecture (*53*), the QuantaMind model further introduces categorical embeddings for DFT methodology, capturing functional and basis set combinations, making it compatible with DFT data from various sources. The architecture is summarized in fig. S12.

Given a system with *N* atoms, input features include nuclear degrees of freedom (atomic numbers $\left\{Z_{1},\ldots ,Z_{N}\right\}$ and coordinates $\left\{r_{1},\ldots ,r_{N}\right\}$), electronic degrees of freedom (total charge Q∈ℤ and spin multiplicity $S\in {\mathbb{N}}_{0}$), and the DFT method encoded as a categorical variable $M\in {\mathbb{N}}_{0}$.

#### Initial embeddings

Each atom *i* is assigned an embedding vector

$$h_{i}^{0}=e_{Z,i}+e_{Q,i}+e_{S,i}+e_{M,i}$$ (4)

where each $e_{\left(\cdot \right),i}\in {\mathbb{R}}^{F}$ is obtained from the respective embedding layers (defined below). These embeddings incorporate atomic identity, system charge, spin state, and DFT method into the learned feature vectors.

#### Interaction layers

Embeddings propagate through three stacked interaction layers. Each layer takes atomic embeddings and relative positions $r_{\mathrm{ij}}≔r_{j}-r_{i}$ and outputs updated hidden states $\left\{h_{i}^{t+1}\right\}$ and corresponding feature vectors $\left\{f_{i}^{t+1}\right\}$. Final per-atom features are

$$f_{i}=\sum_{T=1}^{3}f_{i}^{T}$$ (5)

#### Energy prediction

Atomic contributions to energy are predicted as

$$E_{i}=\text{output}\left(f_{i}\right)$$ (6)

The total potential energy is given by

$$E_{\text{total}}=\sum_{i=1}^{N}E_{i}+E_{\text{rep}}+E_{\text{ele}}+E_{\text{vdw}}$$ (7)

where *E*_rep_, *E*_ele_, and *E*_vdw_ represent empirical corrections for nuclear repulsion, long-range electrostatics, and dispersion forces. Forces are computed as

$$F_{i}=\frac{\partial E_{\text{total}}}{\partial r_{i}}$$ (8)

#### Embedding layers

The nuclear embedding combines an element-specific descriptor and static embedding

$$e_{Z,i}={\text{Md}}_{Z_{i}}+{\tilde{e}}_{Z_{i}}$$ (9)

where $d_{Z_{i}}\in {\mathbb{R}}^{20}$ encodes electronic configuration, **M** is a projection matrix (without bias), and ${\tilde{e}}_{Z_{i}}\in {\mathbb{R}}^{F}$ is an element-specific embedding obtained by selecting the *Z_i_*th row of an embedding matrix.

Charge, spin, and method embeddings share similar architectures, detailed in fig. S13A. Interaction layers consist of both local (fig. S13C) and nonlocal components (fig. S13D). Residual layers (fig. S13E) are commonly used throughout all layers.

#### Mathematical operations and symbols

The model includes standard neural network operations (e.g., dot products, linear layers, Hadamard products) with softplus and SiLU activation functions. The black square symbol (■) stands for the hidden representation from the previous layer/operation. $k_{+/-}\in {\mathbb{R}}^{F}$, $v_{+/-}\in {\mathbb{R}}^{F}$, $W\in {\mathbb{R}}^{F\times F}$, $b\in {\mathbb{R}}^{F}$, $G_{s/p/d}\in {\mathbb{R}}^{F\times K}$, $P_{1/2}\in {\mathbb{R}}^{F\times F}$, and $D_{1/2}\in {\mathbb{R}}^{F\times F}$ are independent trainable parameters of the model, where *K* is the total number of basis functions. W■+b represents a matrix multiplication plus a bias term. W■ represents a linear operation without a bias. <■∣■> represents a scalar product over the feature dimension. ⊙ represents the Hadamard product. The Σ in the local interaction layer represents summation over all neighboring atoms.

softplus(■) represents the softplus activation function

softplus(x)=ln[1+exp(x)] (10)

norm(*x*) represents a normalization over the element dimension

$$\text{norm}\left(a_{i}\right)=\frac{a_{i}}{\sum_{j=1}^{N}a_{j}}$$ (11)

σ(■) represents the SiLU (*54*) activation function

$$\sigma \left(x\right)=\frac{\alpha x}{1+\text{exp}\left(-\beta x\right)}$$ (12)

Here, α and β are trainable parameters.

#### Basis functions

Basis functions **g***_s_*, **g***_p_*, and **g***_d_* are defined via radial Bernstein polynomials and angular real spherical harmonics, with cutoff functions ensuring smooth decay near *r*_cut_. The basis functions $g_{s}\left(r_{\mathrm{ij}}\right)\in {\mathbb{R}}^{K}$, $g_{p}\left(r_{\mathrm{ij}}\right)\in {\mathbb{R}}^{K\times 3}$, and $g_{d}\left(r_{\mathrm{ij}}\right)\in {\mathbb{R}}^{K\times 5}$ are given by

$${}_{k}g_{l}^{m}=\rho_{k}\left(∥r_{\mathrm{ij}}∥\right)\cdot Y_{l}^{m}\left(r_{\mathrm{ij}}\right)$$ (13)

$$r_{\mathrm{ij}}≔r_{j}-r_{i}$$ (14)

where the radial component ρ*_k_* is defined by

$$\rho_{k}\left(r\right)=b_{k,K-1}\text{[exp}\left(-\gamma r\right)\text{]}\cdot f_{\text{cut}}\left(r\right)$$ (15)

and $b_{k,K-1}$ belongs to Bernstein polynomials (k=0,…,K−1) defined by

$$b_{k,K-1}\left(x\right)=\left(\begin{array}{c} K-1\\ k \end{array}\right)x^{k}{\left(1-x\right)}^{K-1-k}$$ (16)

The hyperparameter *K* determines the total number of radial components. The cutoff function is defined by

$$f_{\text{cut}}\left(r\right)=\left\{\begin{array}{ll} \text{exp}\left[-\frac{r^{2}}{\left(r_{\text{cut}}-r\right)\left(r_{\text{cut}}+r\right)}\right] & r<r_{\text{cut}}\\ 0 & r\geq r_{\text{cut}} \end{array}\right.$$ (17)

It ensures that basis functions smoothly decay to zero for $r\geq r_{\text{cut}}$ so that no discontinuities are introduced when atoms enter or leave the cutoff radius (*r*_cut_). The angular component $Y_{l}^{m}\left(r_{\mathrm{ij}}\right)$ in Eq. 13 is given by

$$Y_{l}^{m}\left(r_{\mathrm{ij}}\right)=\left\{\begin{array}{ll} \sqrt{2}\cdot \Pi_{l}^{|m|}\left(z\right)\cdot A_{|m|}\left(x,y\right) & m<0\\ \Pi_{l}^{0}\left(z\right) & m=0\\ \sqrt{2}\cdot \Pi_{l}^{m}\left(z\right)\cdot B_{m}\left(x,y\right) & m>0 \end{array}\right.$$ (18)

$$A_{m}\left(x,y\right)=\sum_{p=0}^{m}\left(\begin{array}{c} m\\ p \end{array}\right)x^{p}y^{m-p}\text{sin}\left[\frac{\pi }{2}\left(m-p\right)\right]$$ (19)

$$B_{m}\left(x,y\right)=\sum_{p=0}^{m}\left(\begin{array}{c} m\\ p \end{array}\right)x^{p}y^{m-p}\text{cos}\left[\frac{\pi }{2}\left(m-p\right)\right]$$ (20)

$$\Pi_{l}^{m}\left(z\right)=\sqrt{\frac{\left(l-m\right)!}{\left(l+m\right)!}}\sum_{p=0}^{\left\lfloor \left(l-m\right)/2\right\rfloor }c_{\mathrm{plm}}r^{2p-l}z^{l-2p-m}$$ (21)

$$r≔∥r_{\mathrm{ij}}∥$$ (22)

where *x*, *y*, and *z* are the three components of **r***_ij_*. Note that the $Y_{l}^{m}$ in Eq. 18 differs from the real form of spherical harmonics by a factor of $\sqrt{\left(4\pi \right)/\left(2l+1\right)}$.

#### Readout layer

Atomic energy predictions are generated via

$$E_{i}=W_{\text{output}}f_{i}+E_{Z_{i}}^{\text{shift}}$$ (23)

where the readout weights $W_{\text{output}}\in {\mathbb{R}}^{1\times F}$ and species-specific energy shifts $E_{Z_{i}}^{\text{shift}}\in \mathbb{R}$ are both trainable.

#### Electrostatics

Partial atomic charges are estimated from features using

$$q_{i}=W_{\text{charge}}f_{i}+q_{Z_{i}}^{\text{shift}}+\frac{1}{N}\left[Q-\sum_{j=1}^{N}\left(W_{\text{charge}}f_{j}+q_{Z_{i}}^{\text{shift}}\right)\right]$$ (24)

ensuring total charge conservation ($\sum_{i}q_{i}=Q$). These charges are used to compute electrostatic energy

$$E_{\text{ele}}=k_{e}\sum_{i}\sum_{j>i}q_{i}q_{j}\left[f_{\text{switch}}\left(r_{\mathrm{ij}}\right)\frac{1}{\sqrt{r_{\mathrm{ij}}^{2}+1}}+\frac{1-f_{\text{switch}}\left(r_{\mathrm{ij}}\right)}{r_{\mathrm{ij}}}\right]$$ (25)

with a smooth switching function $f_{\text{switch}}\left(r\right)$ interpolating between damped and Coulombic regimes

$$f_{\text{switch}}\left(r\right)=\frac{\sigma \left(1-\frac{r-r_{\text{on}}}{r_{\text{off}}-r_{\text{on}}}\right)}{\sigma \left(1-\frac{r-r_{\text{on}}}{r_{\text{off}}-r_{\text{on}}}\right)+\sigma \left(\frac{r-r_{\text{on}}}{r_{\text{off}}-r_{\text{on}}}\right)}$$ (26)

$$\sigma \left(x\right)=\left\{\begin{array}{ll} \text{exp}\left(-\frac{1}{x}\right) & x>0\\ 0 & x\leq 0 \end{array}\right.$$ (27)

with $r_{\text{on}}=0.25\;r_{\text{cut}}$ and $r_{\text{off}}=0.75\;r_{\text{cut}}$.

#### Nuclear repulsion

Short-range repulsion is modeled using a Ziegler-Biersack-Littmark–type potential (*55*)

$$E_{\text{rep}}=k_{e}\sum_{i}\sum_{j>i}\frac{Z_{i}Z_{j}}{r_{\mathrm{ij}}}f_{\text{cut}}\left(r_{\mathrm{ij}}\right)\left[\sum_{k=1}^{4}c_{k}e^{-a_{k}r_{\mathrm{ij}}\left(Z_{i}^{p}+Z_{j}^{p}\right)/d}\right]$$ (28)

Here, *k_e_* is the Coulomb constant and $a_{k},c_{k},p$, and *d* are trainable parameters.

#### Dispersion

Long-range van der Waals interactions are incorporated using a two-body D4 dispersion model (*56*)

$$E_{\text{vdw}}=-\sum_{i}\sum_{j>i}\sum_{n=6,8}s_{n}\frac{C_{\left(n\right)}^{\mathrm{ij}}}{r_{\mathrm{ij}}^{n}}f_{\text{damp}}^{\left(n\right)}\left(r_{\mathrm{ij}}\right)$$ (29)

with trainable D4 parameters and pairwise dispersion coefficients derived from model-predicted partial charges. Unlike the original D4 method, QuantaMind replaces charge equilibration with direct charge prediction.

### QuantaMind model training

Model training is carried out using the PyTorch Lightning framework (*57*). The learning objective is to minimize the root mean squared error of energy, force, and dipole moment predictions. The loss function is defined by

$$L=\alpha_{E}L_{E}+\alpha_{F}L_{F}+\alpha_{\mu }L_{\mu }$$ (30)

where α_E_, α_F_, and α_μ_ are weighting hyperparameters for the energy, force, and dipole losses, respectively. In standard training of QuantaMind, α_E_ = α_F_ = α_μ_ = 1. When comparing with the SO3LR model, α_E_ = 0, α_F_ = 10, and α_μ_ = 1. The energy, force, and dipole loss terms are defined by

$$L_{E}=\sqrt{\frac{1}{B}\sum_{b=1}^{B}{\left(E_{\text{total},b}-E_{\text{total},b}^{\text{ref}}\right)}^{2}}$$ (31)

$$L_{F}=\sqrt{\frac{1}{3N_{\text{atoms}}^{\text{batch}}}\sum_{i=1}^{N_{\text{atoms}}^{\text{batch}}}{\left\|F_{i}-F_{i}^{\text{ref}}\right\|}^{2}}$$ (32)

$$L_{\mu }=\sqrt{\frac{1}{3B}\sum_{b=1}^{B}{\left\|\mu_{b}-\mu_{b}^{\text{ref}}\right\|}^{2}}$$ (33)

The dipole moment of a system is calculated from the predicted partial atomic charges *q_i_* (Eq. 24) by

$$\mu =\sum_{i=1}^{N}q_{i}r_{i}$$ (34)

The model is trained in mini-batches, and *B* is the batch size. If the model is trained from scratch, then *B* is set to 32. If the model is fine-tuned (typically when an additional QM dataset is added to the training set), then *B* is set to 256. $N_{\text{atoms}}^{\text{batch}}$ is the total number of atoms in the mini-batch. The model is trained with the AMSGrad variant of the Adam optimizer (*58*), using a starting learning rate of 0.001. If the evaluation loss does not improve within 25 epochs, then the learning rate is reduced by a factor of 10. Additionally, stochastic weight averaging (*59*) is used to improve generalization.

The number of samples used for model validation and testing is summarized in table S1, and the remaining data points are used for model training. Random splitting is used when partitioning the datasets. Because the datasets vary in size by several orders of magnitude, an oversampling strategy is used during training to ensure the model has an equal opportunity to learn from examples across datasets (*60*). The weights are set to 1, 20, 240, 240, and 240 for the GEMS bottom-up, Crambin top-down, TS1, TS2, and TS3 datasets, respectively.

### TS dataset construction

The TS datasets used in this work were derived from QM cluster simulations (*61*). Three datasets—TS1, TS2, and TS3—were created to represent distinct enzyme-catalyzed reaction pathways and broaden the coverage of TS configurations. These datasets were originally generated as part of independent research projects and not specifically designed for the purpose of training QuantaMind.

TS1 focuses on the enzymatic activity of phenylalanine ammonia-lyase (*62*), which catalyzes the conversion of l-phenylalanine into *trans*-cinnamic acid and ammonia. In addition to l-phenylalanine, we explored alternate substrates, including l-tyrosine and l-lysine, to expand chemical diversity. Crystal structures with Protein Data Bank (PDB) IDs 1 W27, 1Y2M, 6RGS, 6HQF, 6F6T, and 6H2O were used as templates, and ligand conformations were predicted using AutoDock Vina (*63*).

TS2 targets d-psicose 3-epimerase (*64*), initiating from crystal structures with PDB IDs 3VNK, 3VNJ, and 8XIV. Similarly, TS3 explores epoxide hydrolases and their associated enzymatic reactions (*65*), using structures with PDB IDs 5AIF, 5AIG, 5AIH, and 5AII. Native reactant conformations not present in the crystal structures, such as (*p-*nitrophenyl)oxirane, were predicted using AutoDock Vina.

For all three systems, TS searching and structure optimization were conducted using the Gaussian 16 package (*66*) at the B3LYP/def2-SVP level of theory. Each frame obtained during the TS optimization, along with the corresponding single-point energy and atomic forces, was included in the dataset. TS1 comprises 728 entries, TS2 contains 2045 entries, and TS3 comprises 2513 entries, yielding a total of 5286 TS configurations. As noted above, these three datasets were originally generated for independent research projects focused on TS optimization and reaction barrier estimation for the respective enzyme systems; the QM calculations, therefore, followed the standard protocol for those projects. Geometry optimization was performed at the B3LYP/def2-SVP level, which is justified because molecular geometries are less sensitive to basis set size than absolute energies (*67*). Single-point energy calculations at a larger basis set were subsequently carried out on the optimized reactant, product, and TS geometries. However, these single-point calculations yield only a small number of data points per reaction pathway and are, therefore, not suitable for MLFF training. Instead, the training data are drawn from the full optimization trajectory, which provides thousands of geometries with corresponding energies and forces at the B3LYP/def2-SVP level. To quantify the deviation between the two levels of theory, we performed PBE0/def2-TZVPP+MBD single-point calculations on a randomly sampled subset: 17 structures from TS1, 84 from TS2, and 11 from TS3. As shown in fig. S14, the energy parity between B3LYP/def2-SVP and PBE0/def2-TZVPP+MBD is good [Pearson correlation coefficient (*r*) > 0.98]; for the force components, the parity is good at larger magnitudes while smaller-magnitude forces show larger relative deviations. The method embedding layer informs the model that these configurations were calculated at B3LYP/def2-SVP rather than at the primary training level of theory (PBE0/def2-TZVPP+MBD), allowing the model to appropriately distinguish contributions from different levels of theory. As shown in fig. S15, the trained QuantaMind model with method embedding surpasses the B3LYP/def2-SVP reference in force prediction accuracy relative to PBE0/def2-TZVPP+MBD on all three TS datasets, confirming that method embedding and PBE0/def2-TZVPP+MBD training data on other systems together effectively mitigate the level-of-theory mismatch. A full recalculation of the three TS datasets at the PBE0/def2-TZVPP+MBD level would further improve training data quality but is now beyond our computational resources; we identify this as a direction for future work. All other datasets generated in this work for QuantaMind training are calculated at the PBE0/def2-TZVPP+MBD level. The level of theory for each dataset is summarized in table S1.

### HIS, Na^+^, PBS, and PETase dataset construction

The four datasets are generated by sampling configurations from MD simulations. The HIS and PETase datasets are sampled from QuantaMind MD simulations, whereas the Na^+^ and PBS datasets are generated from classical MD trajectories from our internal projects. For the HIS dataset, a histidine molecule capped by residues ACE and NME is solvated in a simulation box with 932 water molecules; an additional bias (similar to the PETase simulation, see the “PETase catalytic cycle simulation” section) is added to the NE─HNE or ND─HND bond to simulate the breaking of these chemical bonds. For the PETase dataset, configurations are sampled from the PETase acylation simulation trajectory. Because each frame from these trajectories contains more than a thousand atoms, far beyond the feasibility of DFT calculations, we used a tailoring algorithm to reduce the model size to fewer than 300 atoms, suitable for QM calculations. The tailoring algorithm is adapted from the “top-down” approach in the GEMS workflow (*8*) to fragment large conformations. Specifically, a point-of-interest (POI) atom is first selected, and the fragment is generated by cutting out a sphere region around that atom. Bond saturation is performed to maintain proper chemical validity where bonds have been severed. The total charge and multiplicity of each fragment are determined on the basis of chemical knowledge in preparation for QM calculations. The algorithm includes the following steps:

1) Identify a POI atom. An initial candidate atom is first selected on the basis of chemical relevance: For the HIS dataset, the candidate is either the NE or the ND atom of histidine, depending on the bias potential used; for the Na^+^ dataset, it is one sodium ion randomly selected from the system; for the PBS dataset, it is the phosphorus atom from one phosphate ion randomly selected from the system; for the PETase dataset, it is the side-chain oxygen atom of the catalytic serine S165. To increase the structural diversity of the extracted fragments, a second atom is then randomly drawn from within a 5-Å radius of this initial candidate and designated as the final POI atom, which serves as the center of the sphere used in the subsequent fragmentation step.

2) Select all atoms within a 5-Å radius of the POI. Chemical bonds are truncated at the boundary of this sphere.

3) Saturate truncated bonds. For each single bond cut at the boundary, if the retained fragment includes a heavy atom (non-hydrogen), replace the missing bonded atom with a hydrogen. The hydrogen position is determined on the basis of the original bond orientation and standard single-bond lengths.

4) If a cut bond is not a single bond (e.g., a double or aromatic bond) or the retained fragment is a single hydrogen, then retain the bonded atom and expand the boundary. The algorithm recursively evaluates new terminal bonds until all cut bonds meet the single bond saturation criterion.

After bond cleavage and saturation, each structure is subjected to DFT single-point energy and force evaluation using the Psi4 software package (*68*) at the PBE0/def2-TZVPP+MBD level of theory (*69*, *70*). After filtering for successful calculations, a total of 1780, 3698, 1833, and 226 molecules were retained for the HIS, Na^+^, PBS, and PETase datasets, respectively.

### MD simulations with QuantaMind

Once trained, the QuantaMind model can be used to perform MD simulations by leveraging its predicted atomic forces. Simulations are carried out using the SchNetPack framework (*71*). SchNetPack’s core MD integrator classes are built on PyTorch, enabling the numerical integration of Newton’s equations of motion to be performed on GPU. The QuantaMind model is also implemented in PyTorch, so energy and force evaluations are GPU-accelerated; the entire simulation pipeline, therefore, runs on GPU.

To model a proton transfer process, we manually modify one of the water molecules in the water boxes by adding an excess proton to form a hydronium ion (H_3_O^+^). The system is evolved under periodic boundary conditions within the NVT ensemble using a 0.5-fs time step at 330 K. The selection of 330 K is to compensate for missing nuclear quantum effects (*19*, *26*).

Hydronium and hydroxide ions are identified in the simulation trajectory based on local hydrogen-oxygen distances. Specifically, an oxygen atom is classified as belonging to a hydronium ion if it is bonded to three hydrogen atoms within a 1.2-Å cutoff. Similarly, an oxygen atom is considered part of a hydroxide ion if it has only one hydrogen within the 1.2-Å cutoff. This threshold is chosen empirically, balancing the covalent O─H bond length in neutral water (0.958 Å) and the typical hydrogen bond length (1.97 Å). To quantify the proton’s mobility, we estimated the diffusion coefficient using the Einstein relation

$$D=\frac{1}{6}\frac{d\;\text{MSD}\left(t\right)}{\mathrm{dt}}$$ (35)

where MSD(*t*) denotes the mean squared displacement as a function of time. To obtain multiple diffusion coefficient estimates per trajectory and characterize measurement uncertainty, we use a bootstrapping procedure: For each 1-ns trajectory, nonoverlapping subtrajectory windows of ∼50 ps are extracted, with a 3-ps gap between consecutive segments. Following Chen *et al.* (*26*), who noted that ion diffusion may be considered uncorrelated after a 3-ps interval, this gap ensures statistical independence between estimates. Each window yields one diffusion coefficient estimate via a linear fit to the MSD; windows with a linear fit coefficient of determination (*R*^2^) < 0.9 are discarded. For each of the six box sizes, three independent 1-ns simulations were performed. The finite-size extrapolation follows the approach of Yeh and Hummer (*20*): A linear model is fitted to all pooled estimates as a function of inverse box length 1/*L* using ordinary least squares (OLS), and the infinite-size diffusion coefficient is obtained from the OLS intercept at 1/*L* = 0. Figure 2D shows both the 95% CI (blue dashed lines) and the 95% prediction interval (red dashed lines); the CI is used to report the final estimate.

For the simulation of water autoionization, the system is evolved under periodic boundary conditions within the NVT ensemble using a 0.5-fs time step at an elevated temperature of 373 K. At each frame, we quantify the number of hydronium and hydroxide ions by evaluating the distances between oxygen and hydrogen atoms, based on geometric criteria consistent with proton transfer. The concentration of hydronium ions, denoted as [H^+^], is calculated by

$$\left[H^{+}\right]=\frac{1}{F}\sum_{f=1}^{F}\frac{N_{H_{3}O^{+}/{\text{OH}}^{-},f}}{N_{A}\times V_{\text{box}}}$$ (36)

where *F* is the total number of frames in the trajectory, *N*_A_ = 6.022 × 10^23^ mol^−1^ is the Avogadro constant, and *V*_box_ is the volume of the simulation box. Accordingly, the estimated pH is calculated by

$$\text{pH}=-{\text{log}}_{10}\left([H^{+}]\right)$$ (37)

For simulations involving acid-base neutralization, we use the SPC216 water box (*72*), a preequilibrated configuration commonly applied in GROMACS simulations. We manually modify one of the water molecules by adding an excess proton to form H_3_O^+^, and a second water molecule is selected ∼10 Å away and modified to form OH^−^ by removing one hydrogen atom.

To estimate the rate constant of the neutralization event, we apply survival analysis (*31*), a statistical framework originally developed in clinical research to model time-to-event data. The objective of survival analysis is to establish a connection between covariates and the time at which an event of interest occurs. A key feature that distinguishes survival analysis from standard regression is its ability to handle right-censored observations: cases in which the event has not yet occurred by the end of the observation window, so only a lower bound on the event time is known. Our neutralization simulation data exhibit exactly this structure: Across 100 independent 50-ps trajectories, neutralization is observed in only a subset of runs; for the remaining trajectories, no reaction occurs within the simulation window, making those observations right-censored. Discarding these censored runs would bias the rate estimate; survival analysis correctly incorporates them.

Let *T* denote a nonnegative random variable representing the reaction time. The survival function *S*(*t*) = *P*(*T* > *t*) gives the probability that the reaction has not yet occurred by time *t*. We fit *S*(*t*) using four parametric models—Weibull, lognormal, log-logistic, and exponential—and select among them by visual inspection of quantile-quantile plots comparing the empirical cumulative distribution function to each fitted model (fig. S16). The lognormal model provides the best fit and is used for all subsequent calculations. We note that, if the exponential model was used instead, then this framework resembles Helfand’s hazard analysis (*73*, *74*), which has been successfully applied to rare-event rate estimation in MD simulations. However, Helfand’s framework was originally developed for transitions in a bistable potential, whereas acid-base neutralization is a diffusion controlled, essentially irreversible process with a very small energy barrier. This physical difference likely explains why the exponential model yields a poorer fit compared to the lognormal model in our case.

The rate constant is then estimated as

$$k=\int_{0}^{+\infty }\frac{N_{\text{reaction}}}{T_{\text{reaction}}\cdot \left[H^{+}\right]}\rho \left(T_{\text{reaction}}\right)\;{\mathrm{dT}}_{\text{reaction}}$$ (38)

where the density $\rho \left(T_{\text{reaction}}\right)$ is calculated from the fitted survival function by

$$\rho \left(T_{\text{reaction}}\right)=-\frac{\mathrm{dS}\left(T_{\text{reaction}}\right)}{{\mathrm{dT}}_{\text{reaction}}}$$ (39)

### PETase catalytic cycle simulation

We began with the crystal structure of the LCC variant (PDB ID: 7W44) (*75*), retaining only the protein structure after removing small molecules and solvents. To incorporate the MHET molecule, we used the crystal structure from PDB ID: 7VVE, which includes MHET and a mutant S165A. Specifically, we aligned the backbone atoms of the catalytic triad from PDB 7VVE to PDB 7W44 and applied this transformation matrix to the MHET molecule in 7VVE. The transformed MHET molecule was then processed with OpenBabel (*76*) to add hydrogen atoms.

We used AmberTools (*77*) for further processing of the protein and MHET structure. Initially, we used the pdb4amber tool to clean the PDB file and add any missing hydrogen atoms. Subsequently, tLEaP was used to combine the protein PDB and the MHET molecule, followed by solvation with TIP3P water using a 5-Å buffer distance. The final system contains 17,792 atoms. All atoms in the system, including solvent water molecules, are described explicitly by the QuantaMind MLFF throughout the simulation.

Following system preparation, we optimized the structure using QuantaMind with the Limited-memory Broyden-Fletcher-Goldfarb-Shanno optimizer, performing 1000 optimization steps (*78*). The optimized system was then relaxed with QuantaMind under periodic boundary conditions within the NPT ensemble, using a 1.0-fs time step at 300 K for 100 ps. The last frame of the NPT simulation was used as the starting point for subsequent NVT simulations with bias potentials to explore the reaction pathways.

The QuantaMind MD simulations were conducted using a single Nvidia A100 GPU with 80 GB of memory. For our system comprising ∼18,000 atoms, each 1-ns simulation required ∼20 hours to complete.

To enhance the simulation process, we introduced a biased potential in addition to the PES predicted by QuantaMind. The biased potential is defined as follows

$$U_{\text{bias}}\left(R_{i},R_{j},t\right)=\frac{1}{2}k{\left[∥R_{i}-R_{j}∥-x\left(t\right)\right]}^{2}$$ (40)

Here, *i* and *j* represent the indices of the atom pairs of interest in the system. **R***_i_* and **R***_j_* are the three-dimensional coordinates of the respective atoms. The constant *k* = 10.0 eV/Å^2^ is a strong force constant, ensuring the distance $∥R_{i}-R_{j}∥$ throughout the simulation. The target distance *x*(*t*) evolves according to

$$x\left(t\right)=x_{\text{start}}+\left\lfloor \frac{t}{30\;\text{ps}}\right\rfloor \cdot \frac{x_{\text{end}}-x_{\text{start}}}{60}$$ (41)

Here, *x*_start_ is the initial distance between atoms *i* and *j*, and *x*_end_ is the desired final distance. The values *i*, *j*, and *x*_end_ are determined on the basis of prior chemical knowledge, detailed as follows.

Acylation reaction: *i* and *j* are indices for the oxygen atom of S165 and the carbonyl carbon of the reactant, respectively, with *x*_end_ = 1.45 Å. Biased potential is only applied for the first 1800 ps of the simulation.

Deacylation reaction—stage 1: *i* and *j* are indices for the oxygen atom of the water reactant and the carbonyl carbon of the reactant, respectively, with *x*_end_ = 1.45 Å. Biased potential is only applied for the first 1800 ps of the simulation

Deacylation reaction—stage 2: *i* and *j* are indices for the oxygen atom of S165 and the carbonyl carbon of the reactant, respectively, with *x*_end_ = 3.5 Å. Biased potential is only applied for the first 1800 ps of the simulation

## Supplementary Materials

### The PDF file includes:

Figs. S1 to S17 Table S1 Legends for movies S1 and S2 References
