# Supplementary material for: Bridging the accuracy-speed divide in reactive molecular dynamics with QuantaMind MD
Source: Sci Adv. 2026 Sep 11;12(37):eaeg3595. doi: 10.1126/sciadv.aeg3595 (PMC13564890; doi:10.1126/sciadv.aeg3595)
Supplement: Supplementary file 1 [file sciadv.aeg3595_sm.pdf]

Science Advances
AAAS

Supplementary Materials for

Bridging the accuracy-speed divide in reactive molecular dynamics with QuantaMind MD

Song Xia *et al.*

Corresponding author: Deqiang Zhang, deqiangzhang@moleculemind.com; Jinbo Xu, jinboxu@moleculemind.com

Sci. Adv. **12**, eaeg3595 (2026)
DOI: 10.1126/sciadv.aeg3595

The PDF file includes:

Figs. S1 to S17
Table S1
Legends for movies S1 and S2
References

Other Supplementary Material for this manuscript includes the following:

Movies S1 and S2

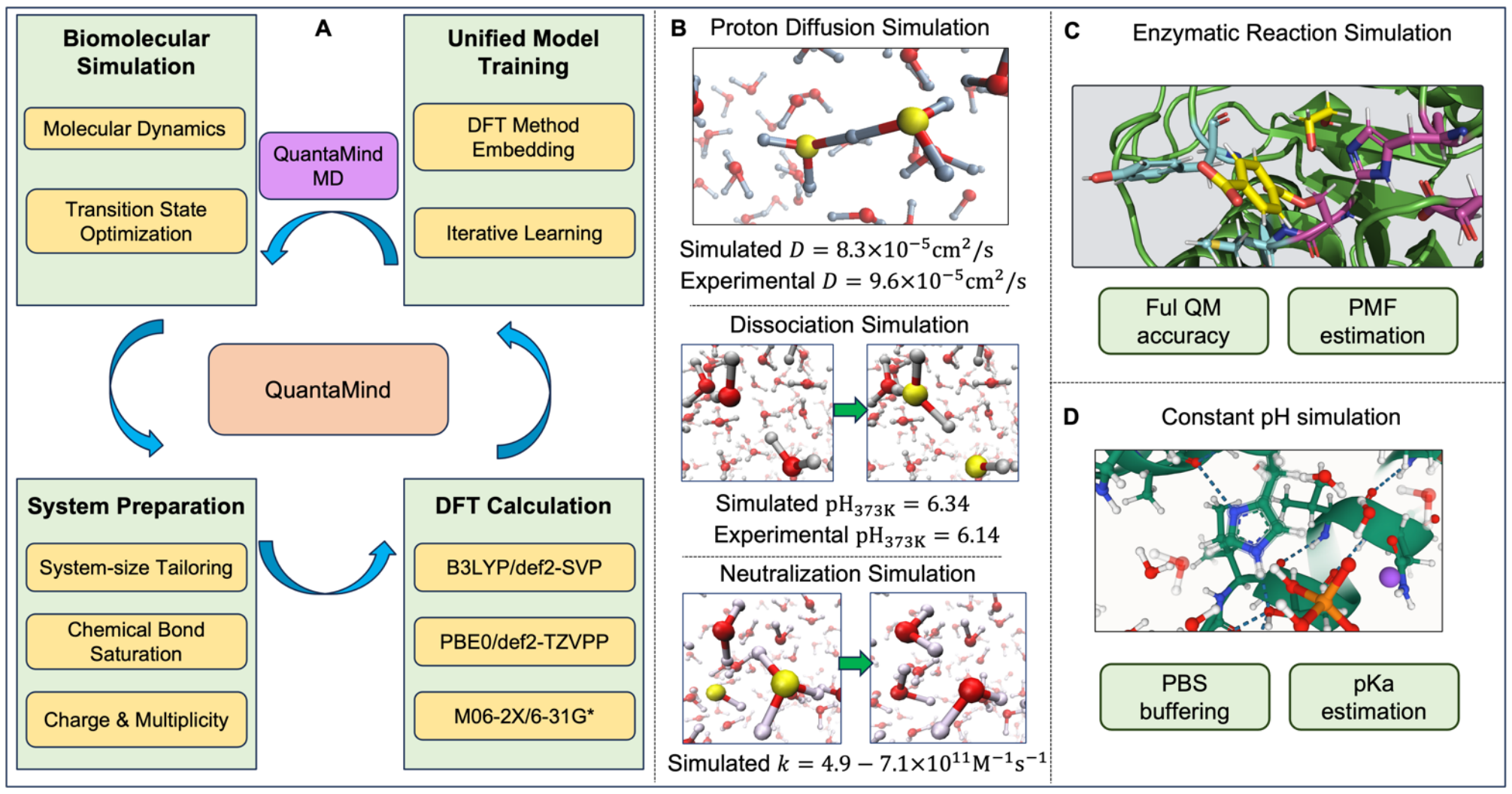

Figure S1: **Development and application of the QuantaMind framework.** (**A**) Overview of the QuantaMind training protocol. (**B**) Simulation of proton diffusion, acid-base neutralization, and water autoionization. (**C**) The PETase enzymatic reaction simulation with full QM accuracy. (**D**) Histidine titration at constant pH with phosphate buffering.

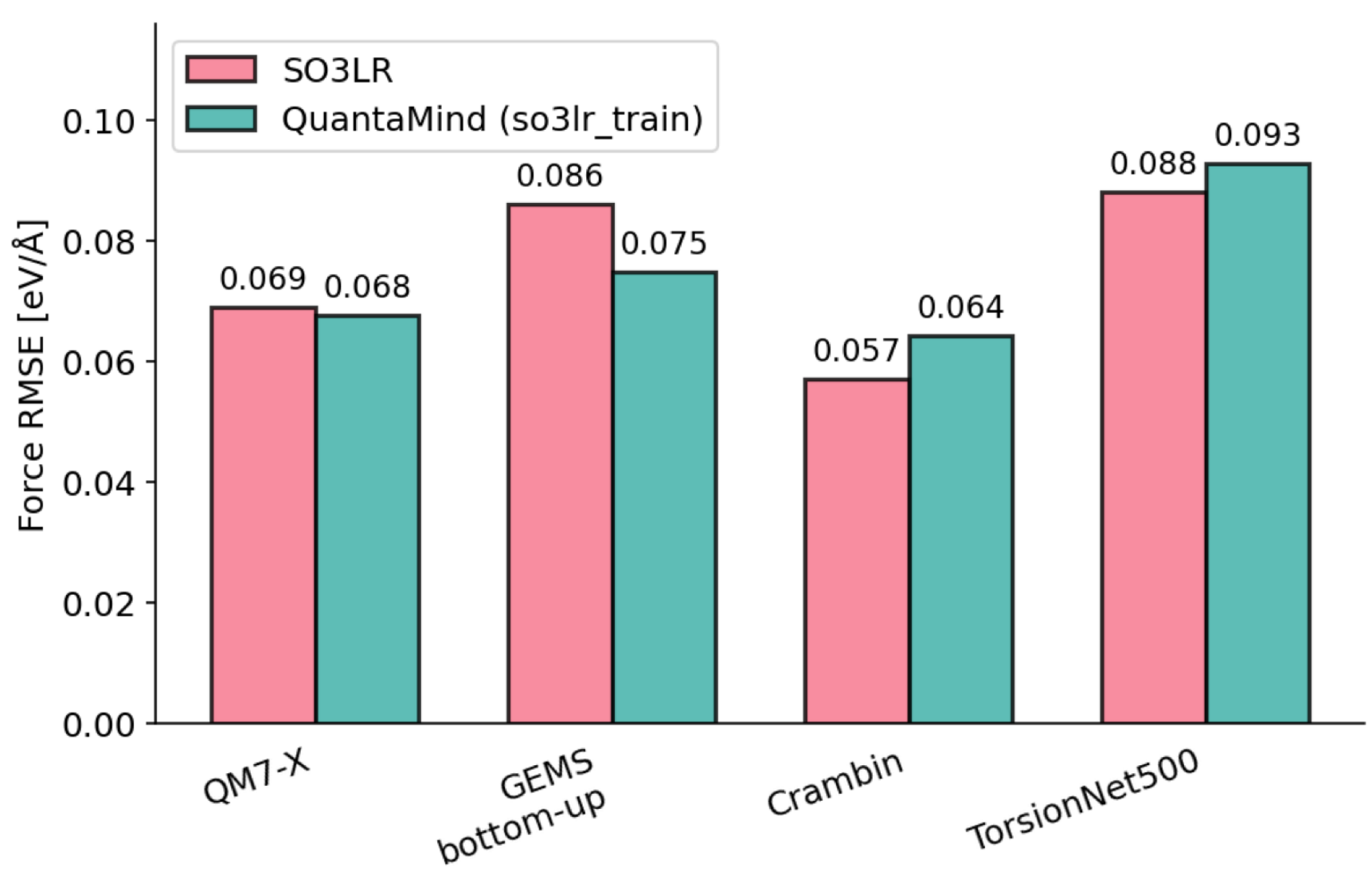

Figure S2: **Model performance comparison on the four benchmark test sets from the SO3LR study.** Force RMSE values for SO3LR are taken from the corresponding research article (*16*). QuantaMind was trained on the SO3LR training data, not on the datasets developed in this work.

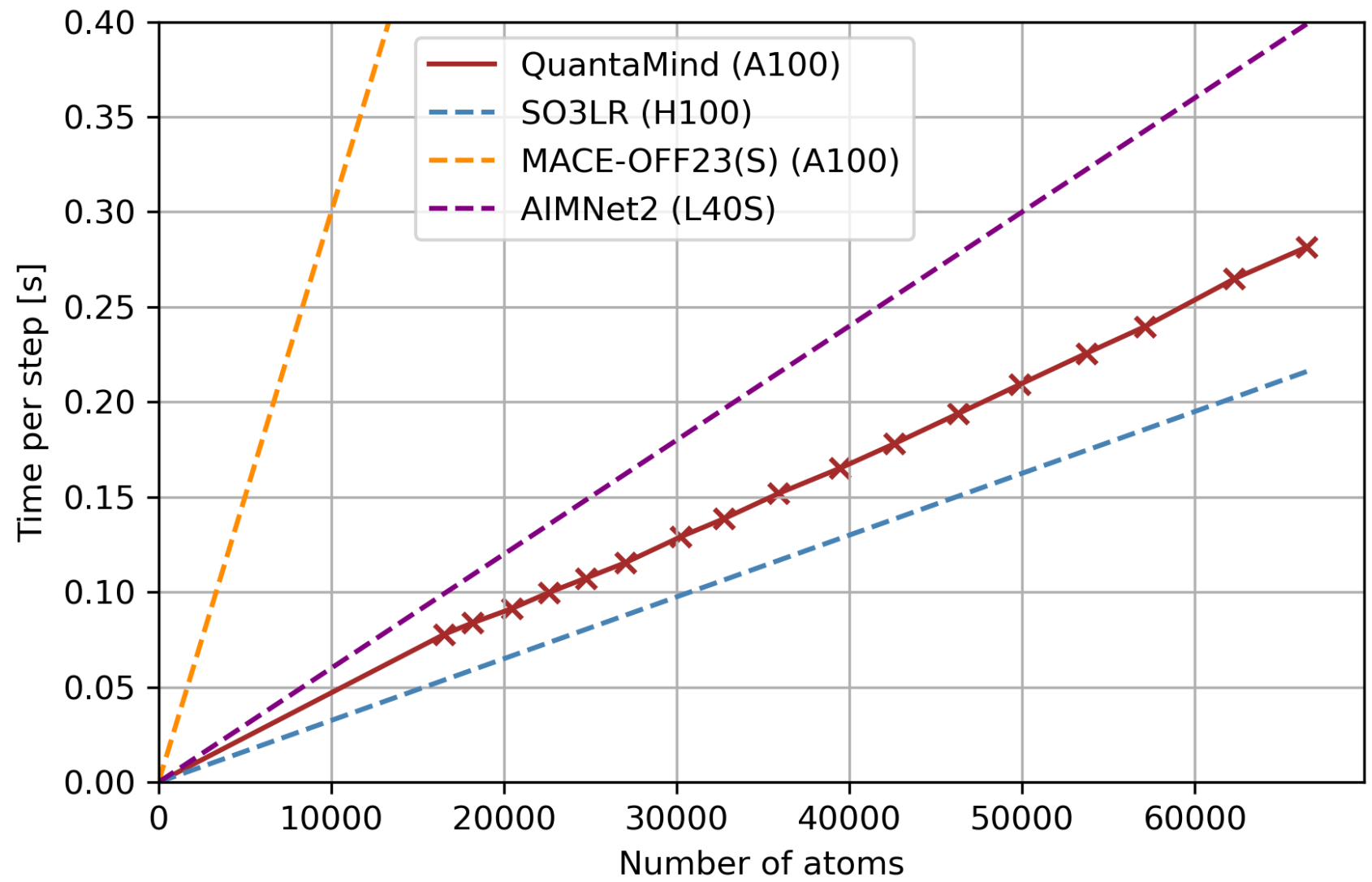

Figure S3: **Inference time per atom per step as a function of system size.** QuantaMind (NVIDIA A100 80GB) is benchmarked on the PETase enzyme system with varying water box sizes prepared using tLeap. The slope for QuantaMind is 4.2×10^{-6} s/atom/step. The slopes for SO3LR (*16*) (NVIDIA H100 80GB), MACE-OFF (*79*) (NVIDIA A100 80GB), and AIMNet2 (*80*) (NVIDIA L40S) are taken from the corresponding articles.

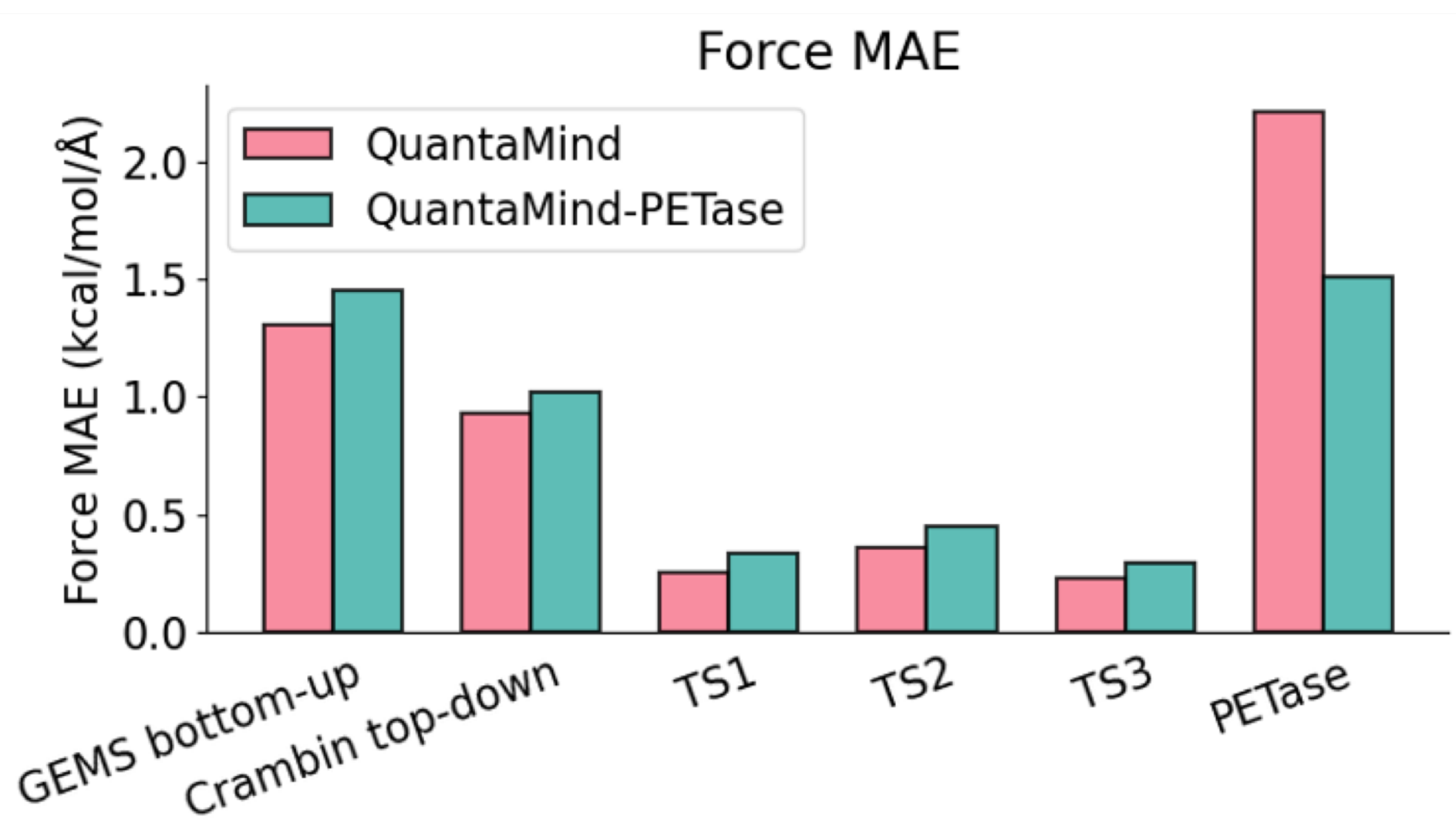

Figure S4: **QuantaMind performance after iterative training on PETase data.** Model performance after adding 226 data points generated from the PETase acylation simulation trajectories and DFT calculation.

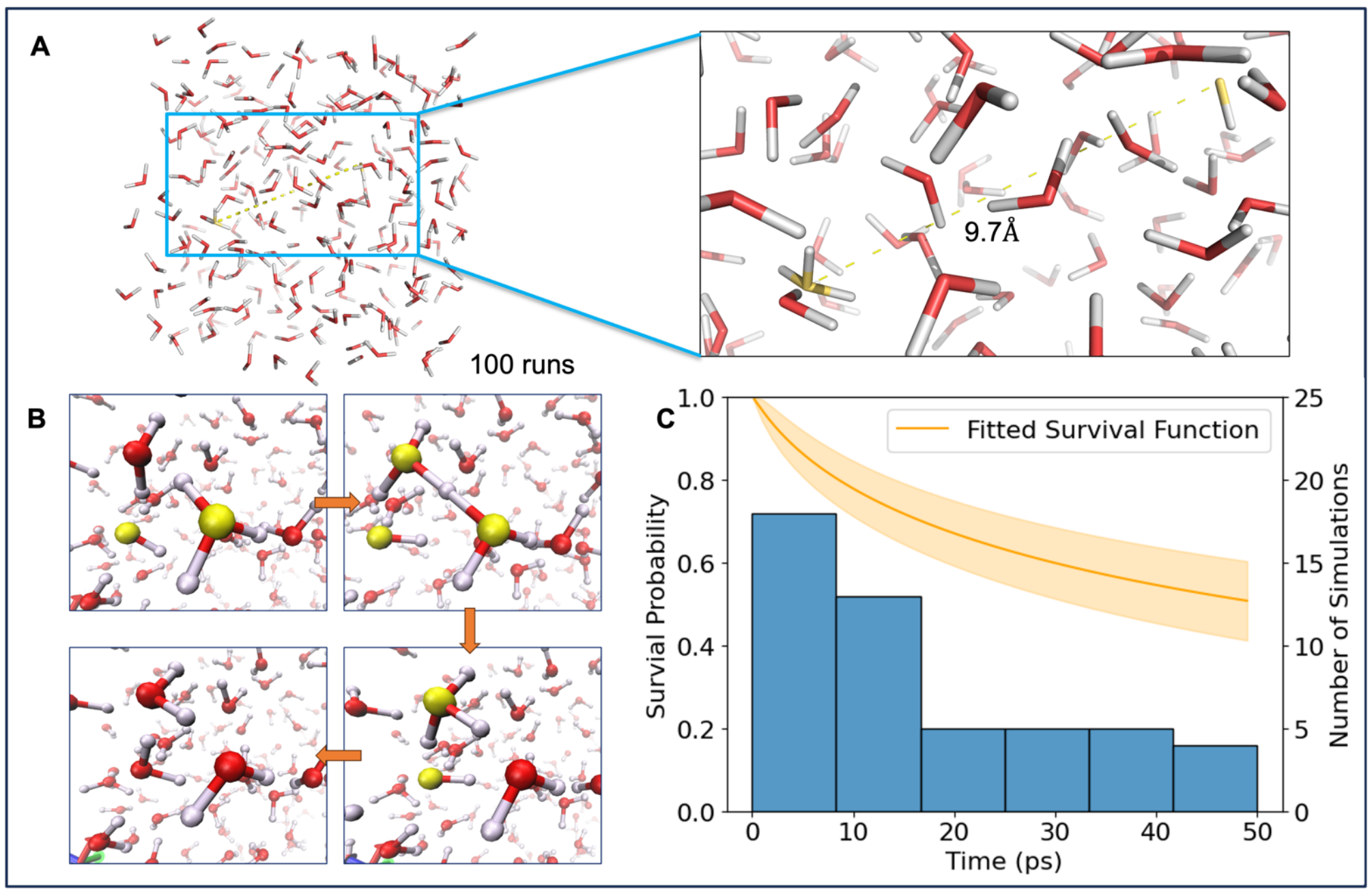

Figure S5: **Simulation of hydronium-hydroxide neutralization in aqueous solution.** (**A**) Initial configuration with H_3O^+ and OH^- ions ~10 Å apart. (**B**) Snapshots of a neutralization event at 12.17 ps in one simulation run. (**C**) Histogram of reaction times across 100 simulations (blue bars) with survival function fit (yellow curve) from survival analysis.

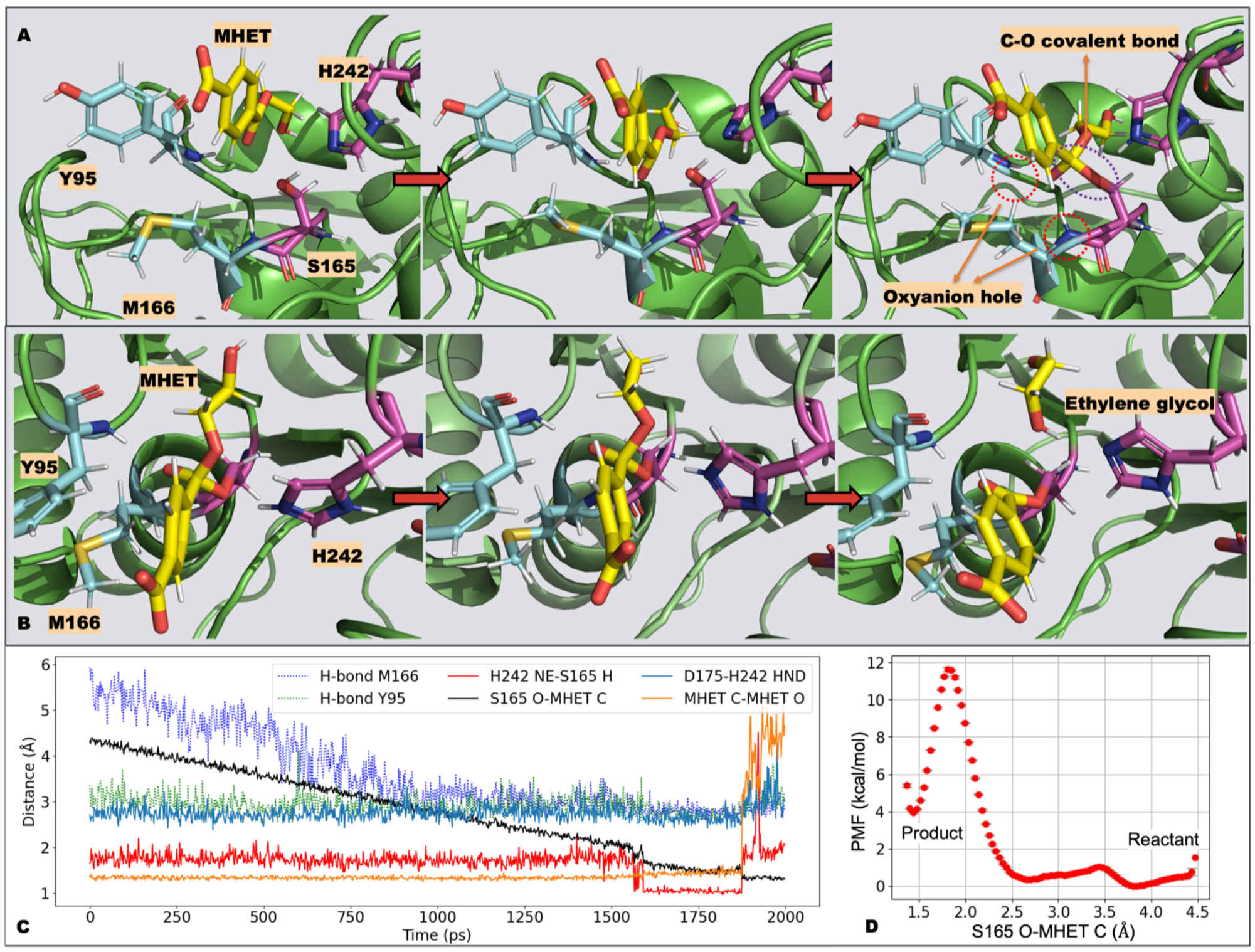

Figure S6: **Simulation of the nucleophilic attack during PETase acylation.** (**A**) Snapshots showing the nucleophilic attack by S165 on the carbonyl carbon of MHET. (**B**) Representative frames showing the carbon-oxygen bond breakage and product release. (**C**) Time series of key interatomic distances: H-bond M166 and H-bond Y95 denote hydrogen bonds in the oxyanion hole; H242 NE–S165 H tracks proton transfer from S165 to H242; S165 O–MHET C is the umbrella sampling coordinate; D175–H242 HND is the closest distance between D175 carboxylate oxygens and the hydrogen on H242's N_{δ}. (**D**) Free energy profile reconstructed using WHAM. Red markers show PMF values, with error bars indicating standard deviations.

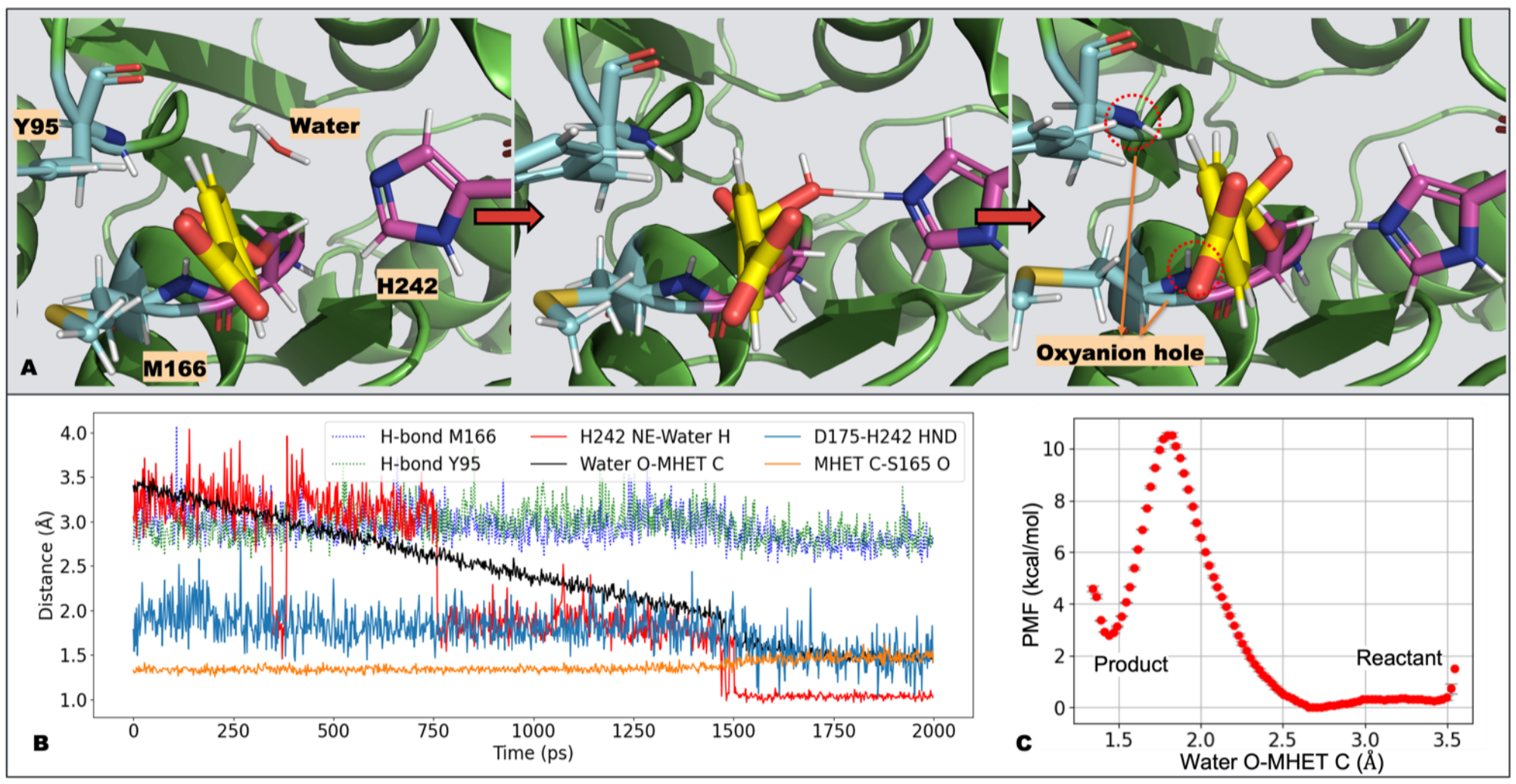

Figure S7: **Simulation of water-mediated nucleophilic attack in PETase deacylation.** (**A**) Representative frames showing the nucleophilic approach of water to the AEI carbonyl carbon. (**B**) Time evolution of key distances: H-bond M166 and H-bond Y95 denote hydrogen bonds in the oxyanion hole; H242 NE–Water H monitors proton transfer from water to H242; MHET C–Water O is the umbrella sampling coordinate; D175–H242 HND is the shortest distance between D175 oxygen atoms and the proton on H242 N_{δ}. (**C**) Free energy profile reconstructed using WHAM. Red markers show PMF values, with error bars indicating standard deviations.

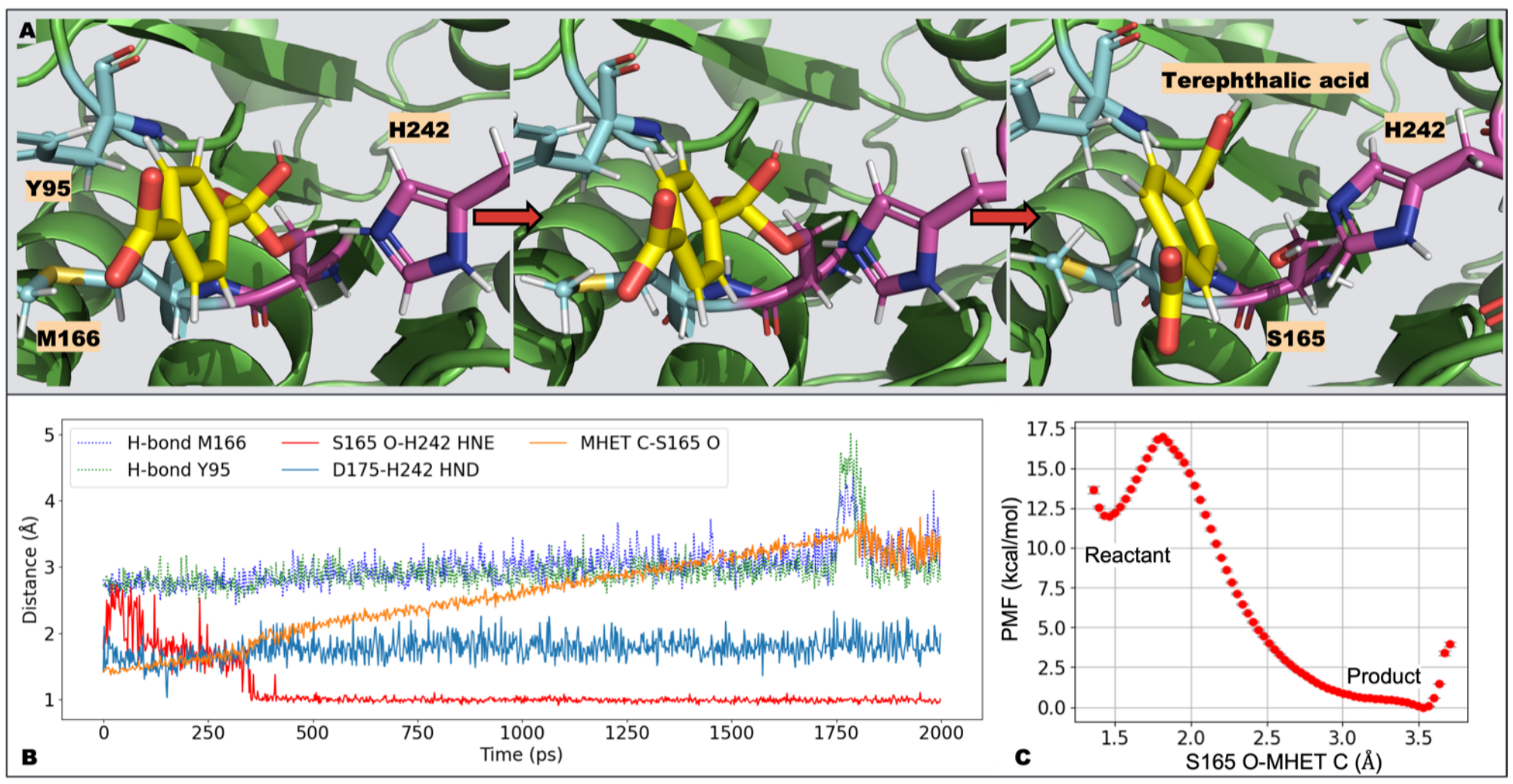

Figure S8: **Simulation of ester bond cleavage and catalytic triad regeneration in PETase deacylation.** (**A**) Visualization of ester bond cleavage, producing terephthalic acid and restoring the active site. (**B**) Monitored interatomic distances: H-bond M166 and H-bond Y95 denote hydrogen bonds in the oxyanion hole; S165 O–H242 HNE tracks proton transfer from H242 to regenerate S165; MHET C–S165 O is the umbrella sampling coordinate; D175–H242 HND is the shortest distance between D175 oxygen atoms and the proton on H242 N_{δ}. (**C**) Free energy profile reconstructed using WHAM. Red markers show PMF values, with error bars indicating standard deviations.

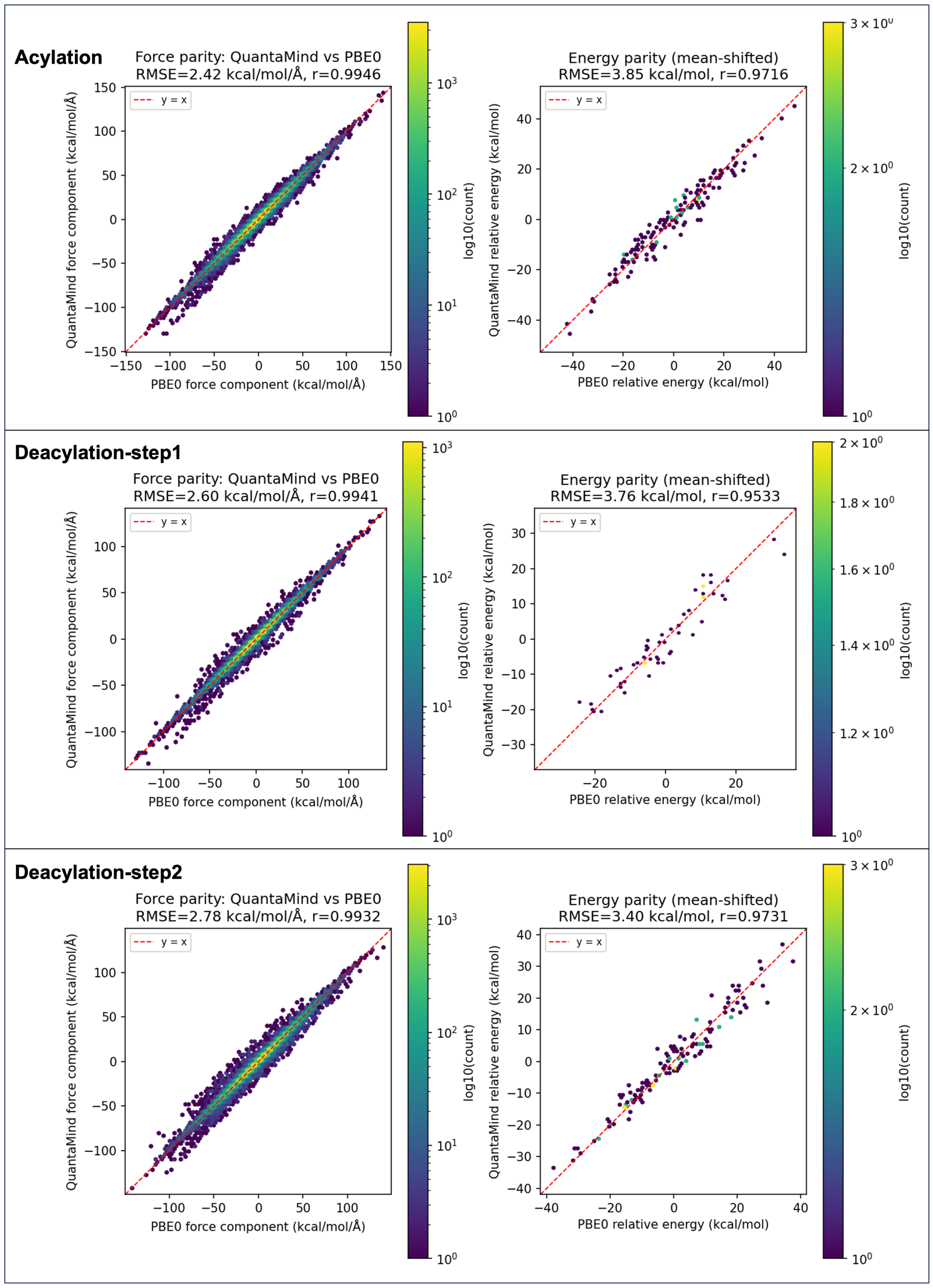

Figure S9: **Validation of QuantaMind against PBE0/def2-TZVPP+MBD on geometries sampled from the PETase simulation.** Force component parity (left column) and energy parity (right column) between QuantaMind predictions and single-point PBE0/def2-TZVPP+MBD calculations on geometries randomly sampled from each stage of the PETase simulation: 151 geometries from the acylation step, 54 from deacylation stage 1, and 137 from deacylation stage 2. The POI atom is the catalytic serine OG. Each panel is a hexbin plot; color indicates the number of data points in each hexagonal bin.

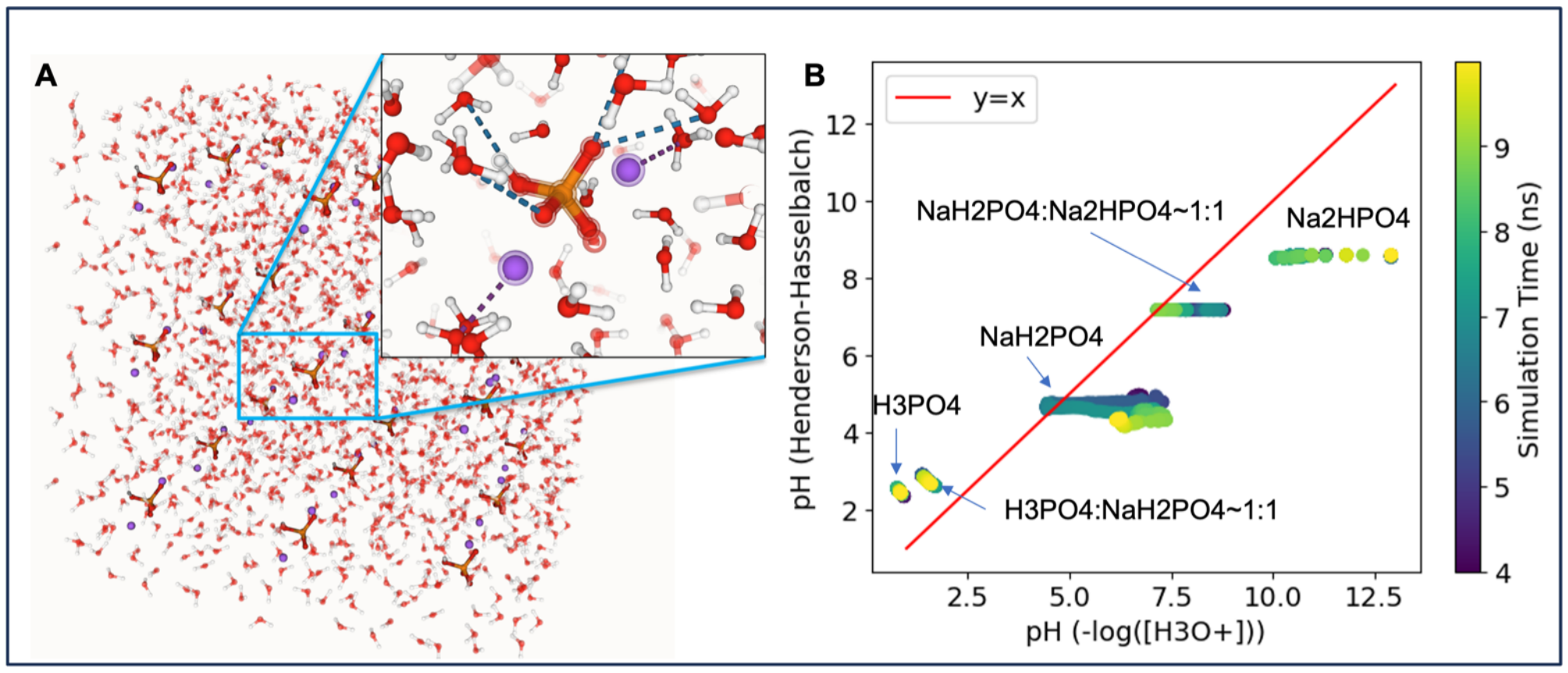

Figure S10: **Simulation of phosphate-buffered saline using QuantaMind-titration.** (**A**) PBS simulation environment with 27 grid sites; each site contains one of the phosphate species: Na_3PO_4, Na_2HPO_4, NaH_2PO_4, or H_3PO_4. (**B**) Relationship between hydronium-concentration pH and Henderson-Hasselbalch pH, across five independent 10 ns simulations. Color gradient reflects simulation time. The red line indicates $y = x$.

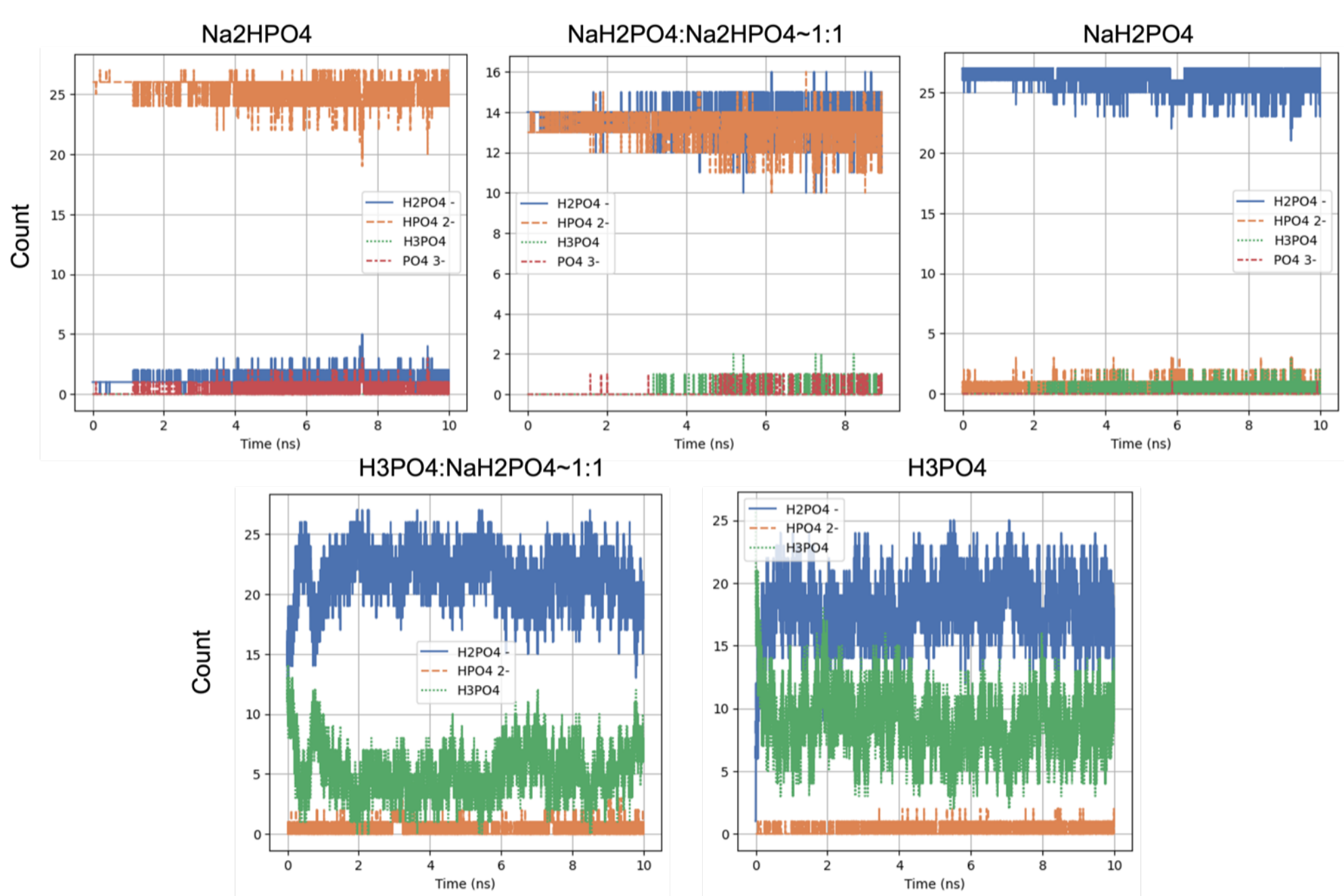

Figure S11: **Species count during PBS simulations.** Species count during the simulations of the 5 PBS systems.

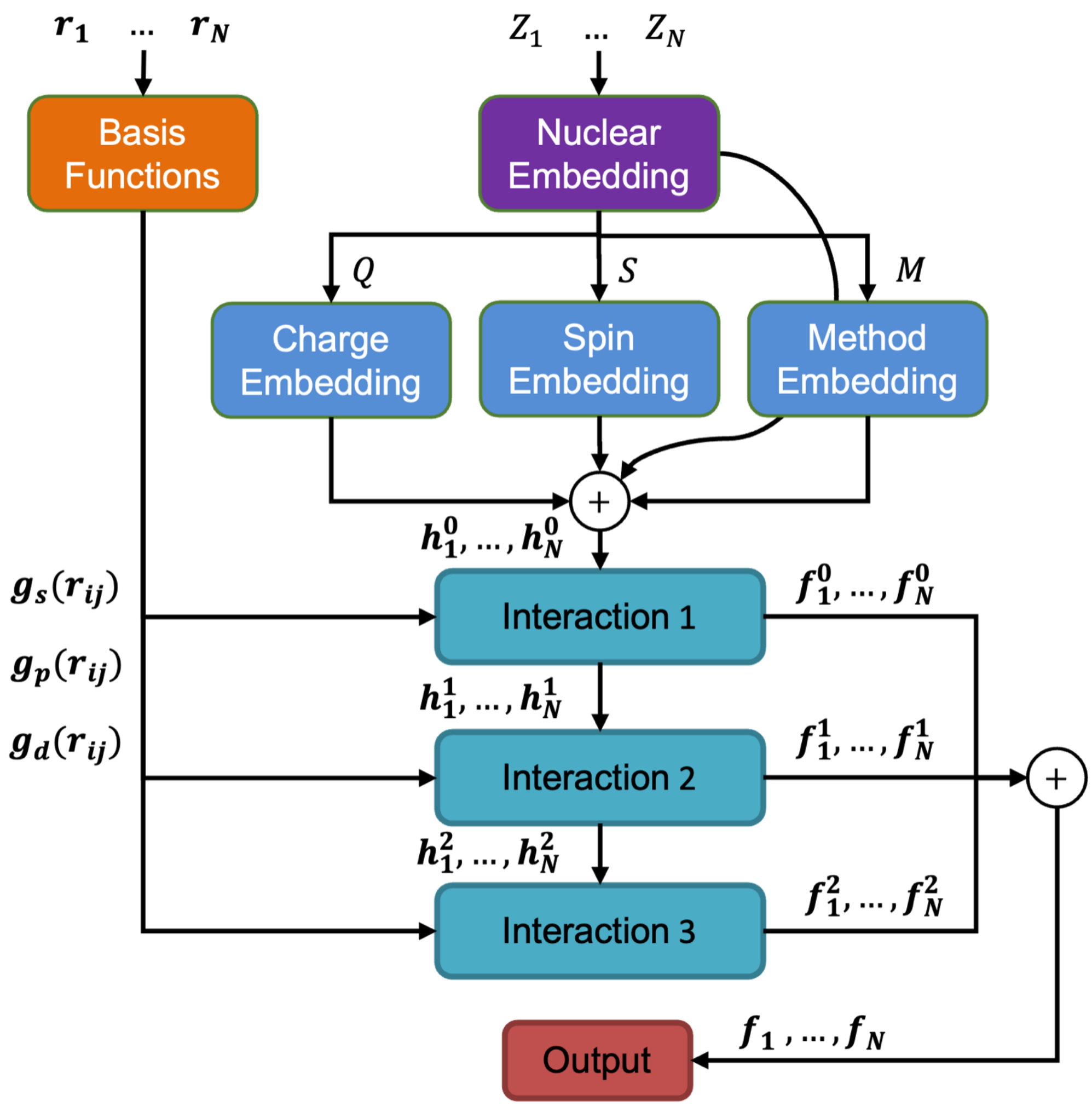

Figure S12: **Architecture of the QuantaMind model.** Details of charge, spin, and method embedding layers, along with interaction layers, are expanded in fig. S13.

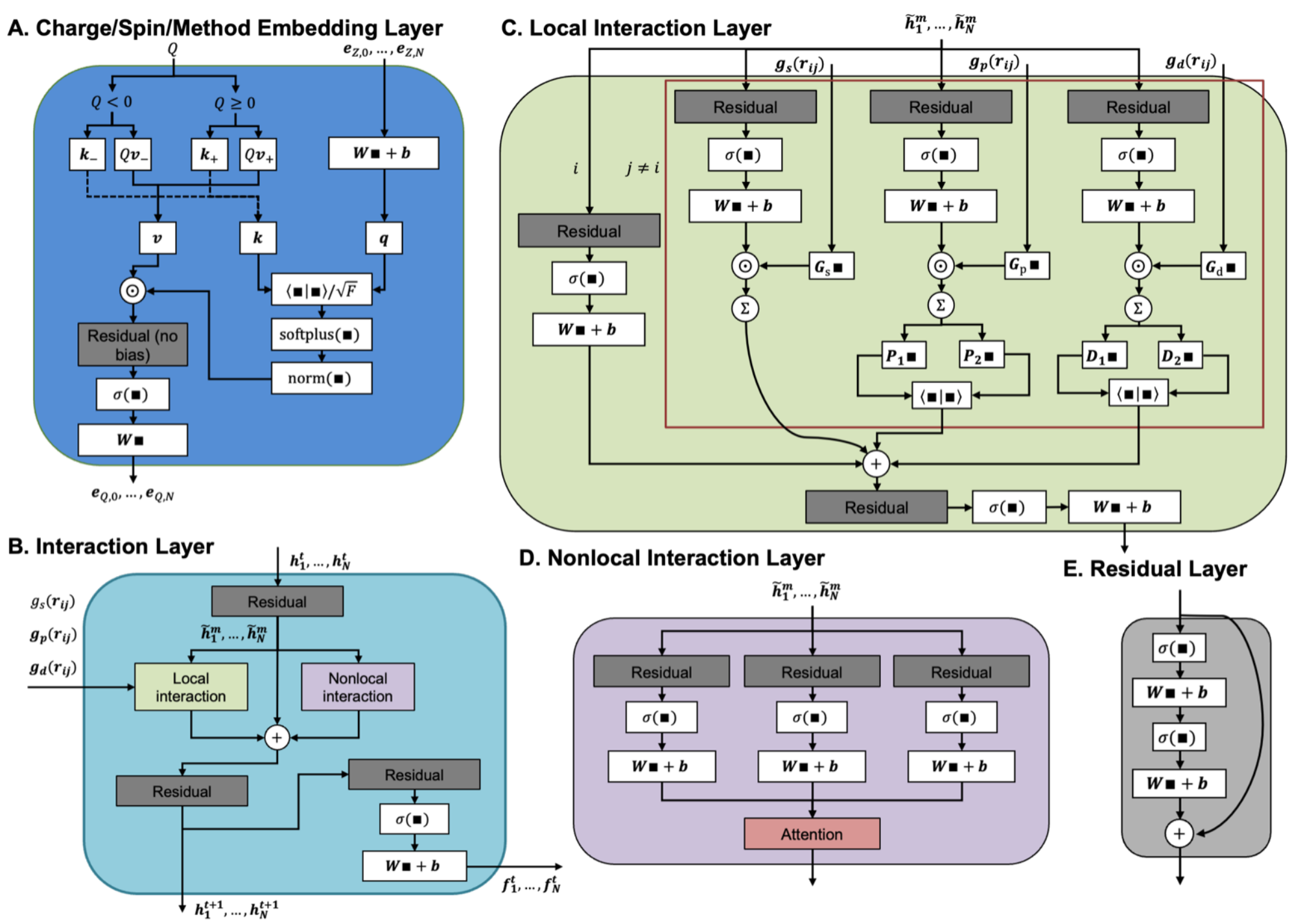

Figure S13: **Detailed structure of embedding and interaction layers.** Symbols and learnable transformations are defined in the accompanying text.

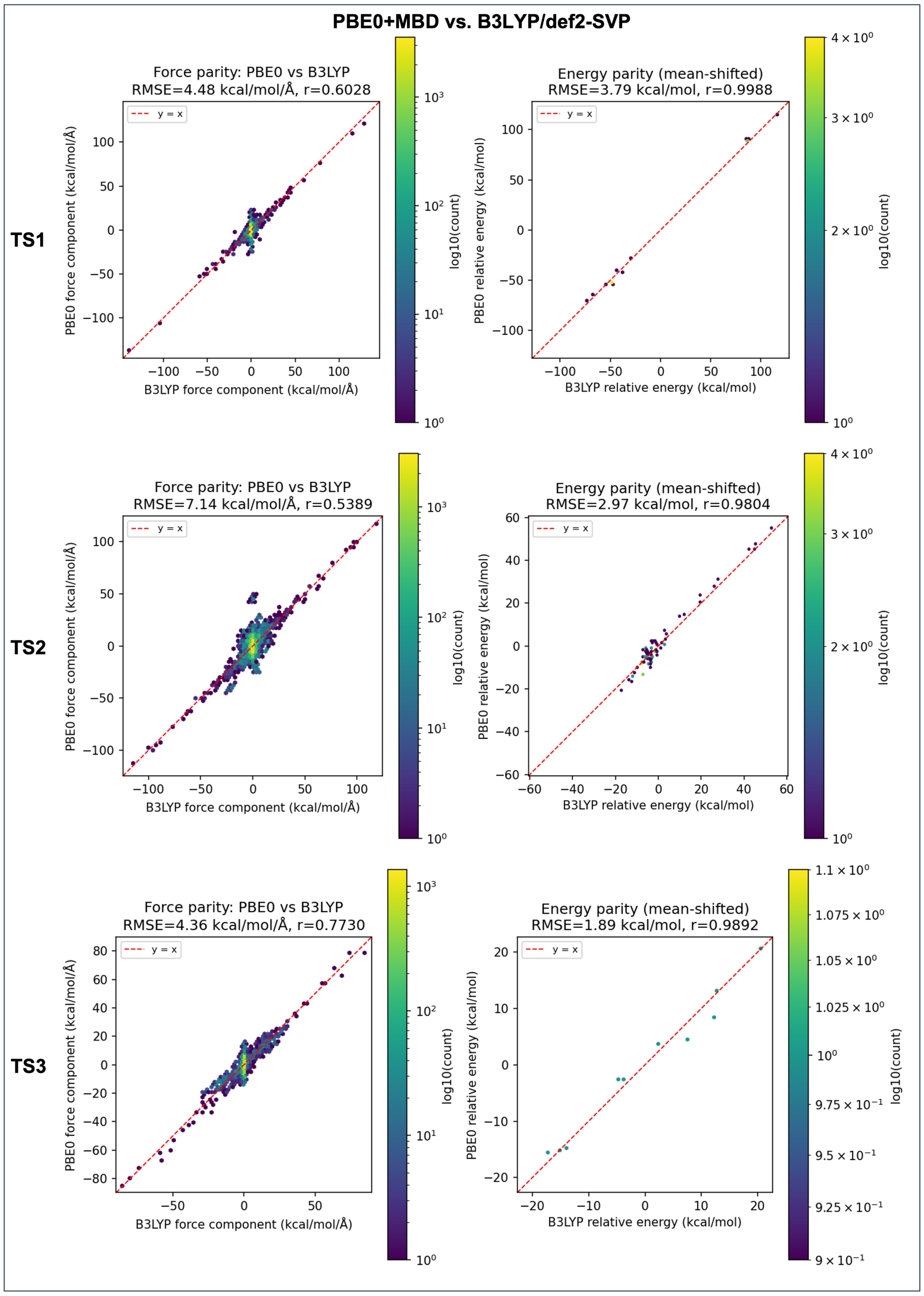

Figure S14: **Comparison of B3LYP/def2-SVP and PBE0/def2-TZVPP+MBD on transition state structures.** Parity plots for energies (left column) and force components (right column) computed at B3LYP/def2-SVP versus PBE0/def2-TZVPP+MBD on a randomly sampled subset of 17, 84, and 11 structures from TS1, TS2, and TS3, respectively. Each panel is a hexbin plot; color indicates the number of data points in each hexagonal bin.

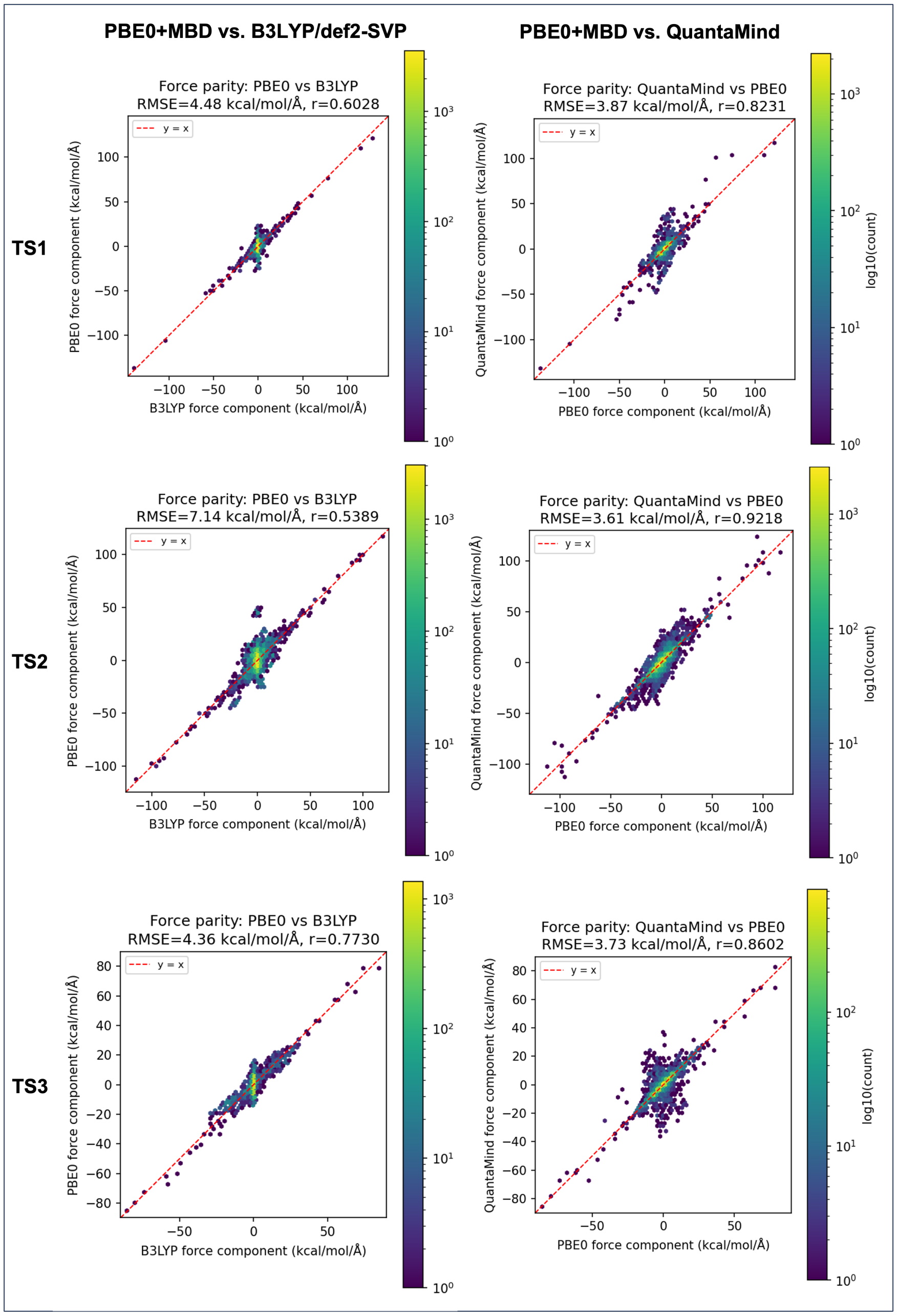

Figure S15: **Method embedding and PBE0+MBD data mitigates the level-of-theory mismatch in the transition state datasets.** Left column: force component parity between B3LYP/def2-SVP and PBE0/def2-TZVPP+MBD on the sampled TS structures. Right column: force component parity between QuantaMind predictions and PBE0/def2-TZVPP+MBD on the same structures. Each panel is a hexbin plot; color indicates the number of data points in each hexagonal bin.

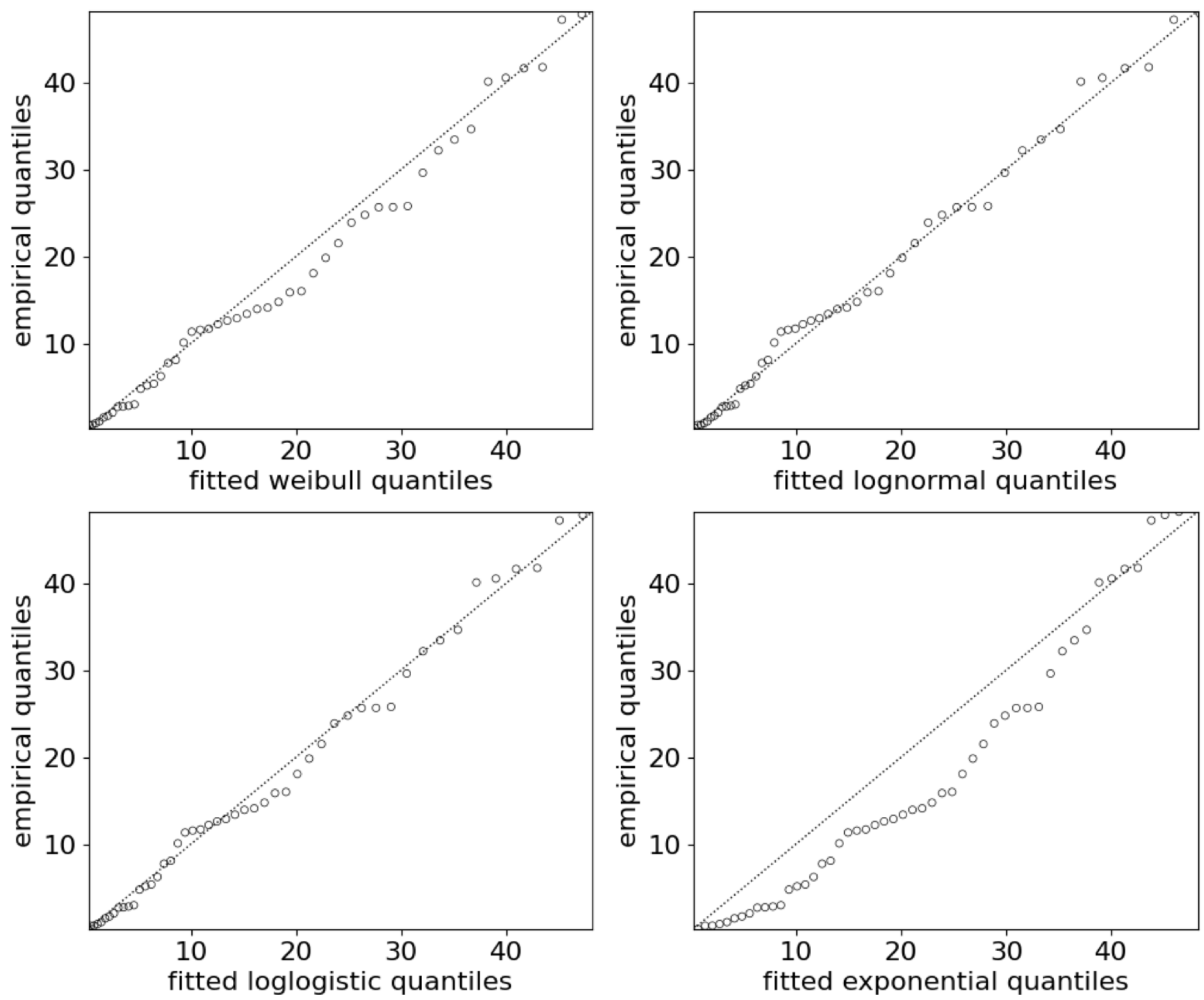

Figure S16: **Quantile-quantile plots for parametric survival function fits.** QQ plots comparing the empirical cumulative distribution function of neutralization reaction times against four candidate parametric models: Weibull, lognormal, log-logistic, and exponential.

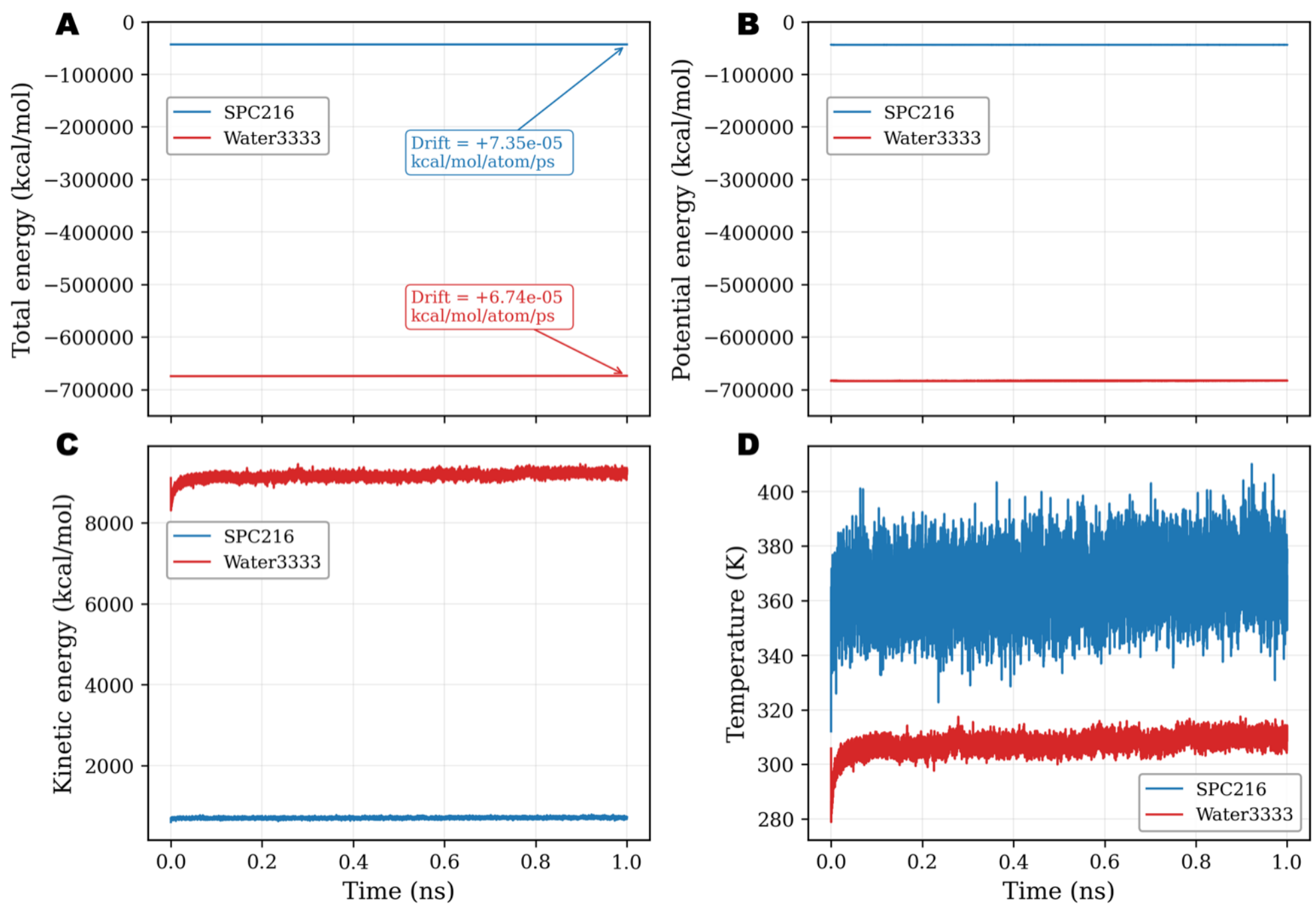

Figure S17: **NVE simulations of pure water systems using QuantaMind.** 1 ns NVE trajectories of the SPC216 system (216 water molecules, blue) and the Water3333 system (3333 water molecules, red). (**A**) Total energy, (**B**) potential energy, (**C**) kinetic energy, and (**D**) instantaneous temperature as a function of simulation time. The total energy drift is $+7.35\times10^{-5}$ kcal/mol/atom/ps for SPC216 and $+6.74 \times 10^{-5}$ kcal/mol/atom/ps for Water3333.

Table S1: **Datasets used in this work.**

Name	# Structures	# Atoms	Elements	# Val/Test	Level of Theory	Source
PETase	226	[92, 146]	H C N O S	20/20	PBE0/def2-TZVPP+MBD	This work
Na^{+}	3,698	[10, 137]	H C N O Na S	300/300	PBE0/def2-TZVPP+MBD	This work
HIS	1,780	[28, 82]	H C N O	90/90	PBE0/def2-TZVPP+MBD	This work
PBS	1,833	[6, 220]	H C N O Na P S Zn	180/180	PBE0/def2-TZVPP+MBD	This work
TS1	727	[264, 269]	H C N O	70/70	B3LYP/def2-SVP	This work
TS2	2,044	115	H C N O Co	70/70	B3LYP/def2-SVP	This work
TS3	2,613	287	H C N O S	70/70	B3LYP/def2-SVP	This work
GEMS bottom-up	2,713,986	[1, 120]	H C N O S	5000/5000	PBE0/def2-TZVPP+MBD	(*8*)
Crambin top-down	5,624	[230, 320]	H C N O S	500/500	PBE0/def2-TZVPP+MBD	(*8*)
SO3LR	2,230,341	[1, 120]	H C N O F S Cl	5000/5000	PBE0/tight+MBD	(*16*)

REFERENCES

1. M. Karplus, G. A. Petsko, Molecular dynamics simulations in biology. *Nature* **347**, 631–639 (1990).

2. M. Karplus, J. A. McCammon, Molecular dynamics simulations of biomolecules. *Nat. Struct. Biol.* **9**, 646–652 (2002).

3. M. S. Gordon, M. W. Schmidt, "Advances in electronic structure theory: GAMESS a decade later," in *Theory and Applications of Computational Chemistry* (Elsevier, 2005), pp. 1167–1189.

4. O. T. Unke, S. Chmiela, H. E. Sauceda, M. Gastegger, I. Poltavsky, K. T. Schutt, A. Tkatchenko, K.-R. Müller, Machine learning force fields. *Chem. Rev.* **121**, 10142–10186 (2021).

5. A. Kabylda, B. Mortazavi, X. Zhuang, A. Tkatchenko, Mechanical properties of nanoporous graphenes: Transferability of graph machine-learned force fields compared to local and reactive potentials. *Adv. Funct. Mater.* **35**, 2417891 (2025).

6. T. Plé, O. Adjoua, A. Benali, E. Posenitskiy, C. Villot, L. Lagardère, J.-P. Piquemal, A foundation model for accurate atomistic simulations in drug design. ChemRxiv [Preprint] (2025). https://doi.org/10.26434/chemrxiv-2025-f1hgn-v3.

7. O. T. Unke, M. Meuwly, PhysNet: A neural network for predicting energies, forces, dipole moments, and partial charges. *J. Chem. Theory Comput.* **15**, 3678–3693 (2019).

8. O. T. Unke, M. Stöhr, S. Ganscha, T. Unterthiner, H. Maennel, S. Kashubin, D. Ahlin, M. Gastegger, L. M. Sandonas, J. T. Berryman, A. Tkatchenko, K.-R. Müller, Biomolecular dynamics with machine-learned quantum-mechanical force fields trained on diverse chemical fragments. *Sci. Adv.* **10**, eadn4397 (2024).

9. T. Wang, X. He, M. Li, Y. Li, R. Bi, Y. Wang, C. Cheng, X. Shen, J. Meng, H. Zhang, H. Liu, Z. Wang, S. Li, B. Shao, T. Y. Liu, Ab initio characterization of protein molecular dynamics with AI2BMD. *Nature* **635**, 1019–1027 (2024).

10. M. Schreiner, A. Bhowmik, T. Vegge, J. Busk, O. Winther, Transition1x-a dataset for building generalizable reactive machine learning potentials. *Sci. Data* **9**, 779 (2022).

11. E. C.-Y. Yuan, A. Kumar, X. Guan, E. D. Hermes, A. S. Rosen, J. Zádor, T. Head-Gordon, S. M. Blau, Analytical ab initio hessian from a deep learning potential for transition state optimization. *Nat. Commun.* **15**, 8865 (2024).

12. D. M. Anstine, Q. Zhao, R. Zubatiuk, S. Zhang, V. Singla, F. Nikitin, B. M. Savoie, O. Isayev, AIMNet2-rxn: A machine learned potential for generalized reaction modeling on a millions-of-pathways scale. ChemRxiv [Preprint] (2025). https://doi.org/10.26434/chemrxiv-2025-hpdmg.

13. S. Perego, L. Bonati, Data efficient machine learning potentials for modeling catalytic reactivity via active learning and enhanced sampling. *npj Comput. Mater.* **10**, 291 (2024).

14. R. David, M. de la Puente, A. Gomez, O. Anton, G. Stirnemann, D. Laage, ArcaNN: Automated enhanced sampling generation of training sets for chemically reactive machine learning interatomic potentials. *Digit. Discov.* **4**, 54–72 (2024).

15. K. Töpfer, L. I. Vazquez-Salazar, M. Meuwly, Asparagus: A toolkit for autonomous, user-guided construction of machine-learned potential energy surfaces. *Comput. Phys. Commun.* **308**, 109446 (2025).

16. A. Kabylda, J. T. Frank, S. Suárez-Dou, A. Khabibrakhmanov, L. M. Sandonas, O. T. Unke, S. Chmiela, K.-R. Müller, A. Tkatchenko, Molecular simulations with a pretrained neural network and universal pairwise force fields. *J. Am. Chem. Soc.* **147**, 33723–33734 (2025).

17. Y. Wang, K. Takaba, M. S. Chen, M. Wieder, Y. Xu, T. Zhu, J. Z. Zhang, A. Nagle, K. Yu, X. Wang, D. J. Cole, J. A. Rackers, K. Cho, J. G. Greener, P. Eastman, S. Martiniani, M. E. Tuckerman, On the design space between molecular mechanics and machine learning force fields. *Appl. Phys. Rev.* **12**, 021304 (2025).

18. D. Marx, Proton transfer 200 years after von Grotthuss: Insights from Ab initio simulations. *ChemPhysChem* **7**, 1848–1870 (2006).

19. D. Muñoz-Santiburcio, Accurate diffusion coefficients of the excess proton and hydroxide in water via extensive Ab initio simulations with different schemes. *J. Chem. Phys.* **157**, 024504 (2022).

20. I.-C. Yeh, G. Hummer, System-size dependence of diffusion coefficients and viscosities from molecular dynamics simulations with periodic boundary conditions. *J. Phys. Chem. B.* **108**, 15873–15879 (2004).

21. J. Huo, J. Chen, P. Liu, B. Hong, J. Zhang, H. Dong, S. Li, Microscopic mechanism of proton transfer in pure water under ambient conditions. *J. Chem. Theory Comput.* **19**, 4243–4254 (2023).

22. H. Weingärtner, C. A. Chatzidimttriou-Dreismann, Anomalous H^+ and D^+ conductance in H^2O–D^2O mixtures. *Nature* **346**, 548–550 (1990).

23. B. Halle, G. Karlström, Prototropic charge migration in water. Part 2.—Interpretation of nuclear magnetic resonance and conductivity data in terms of model mechanisms. *J. Chem. Soc. Faraday Trans. 2* **79**, 1047–1073 (1983).

24. Y. Wu, H. Chen, F. Wang, F. Paesani, G. A. Voth, An improved multistate empirical valence bond model for aqueous proton solvation and transport. *J. Phys. Chem. B.* **112**, 467–482 (2008).

25. K. Park, W. Lin, F. Paesani, A refined MS-EVB model for proton transport in aqueous environments. *J. Phys. Chem. B.* **116**, 343–352 (2012).

26. M. Chen, L. Zheng, B. Santra, H.-Y. Ko, R. A. DiStasio Jr, M. L. Klein, R. Car, X. Wu, Hydroxide diffuses slower than hydronium in water because its solvated structure inhibits correlated proton transfer. *Nat. Chem.* **10**, 413–419 (2018).

27. A. Grossfield, WHAM: The weighted histogram analysis method, version 2.0.9. http://membrane.urmc.rochester.edu/content/wham [accessed 15 November 2013].

28. A. V. Bandura, S. N. Lvov, The ionization constant of water over wide ranges of temperature and density. *J. Phys. Chem. Ref. Data Monogr.* **35**, 15–30 (2006).

29. P. W. Atkins, J. De Paula, J. Keeler, *Atkins' Physical Chemistry* (Oxford Univ. Press, 2023).

30. A. Hassanali, M. K. Prakash, H. Eshet, M. Parrinello, On the recombination of hydronium and hydroxide ions in water. *Proc. Natl. Acad. Sci. U.S.A.* **108**, 20410–20415 (2011).

31. C. Davidson-Pilon, lifelines: Survival analysis in Python. *J. Open Source Softw.* **4**, 1317 (2019).

32. M. Eigen, L. De Maeyer, Self-dissociation and protonic charge transport in water and ice. *Proc. Royal Soc. Lond. Ser. A Math. Phys. Sci.* **247**, 505–533 (1958).

33. S. Yoshida, K. Hiraga, T. Takehana, I. Taniguchi, H. Yamaji, Y. Maeda, K. Toyohara, K. Miyamoto, Y. Kimura, K. Oda, A bacterium that degrades and assimilates poly (ethylene terephthalate). *Science* **351**, 1196–1199 (2016).

34. H. Lu, D. J. Diaz, N. J. Czarnecki, C. Zhu, W. Kim, R. Shroff, D. J. Acosta, B. R. Alexander, H. O. Cole, Y. Zhang, N. A. Lynd, A. D. Ellington, H. S. Alper, Machine learning-aided engineering of hydrolases for PET depolymerization. *Nature* **604**, 662–667 (2022).

35. V. Tournier, C. Topham, A. Gilles, B. David, C. Folgoas, E. Moya-Leclair, E. Kamionka, M.-L. Desrousseaux, H. Texier, S. Gavalda, M. Cot, E. Guémard, M. Dalibey, J. Nomme, G. Cioci, S. Barbe, M. Chateau, I. André, S. Duquesne, A. Marty, An engineered PET depolymerase to break down and recycle plastic bottles. *Nature* **580**, 216–219 (2020).

36. L. Hedstrom, Serine protease mechanism and specificity. *Chem. Rev.* **102**, 4501–4524 (2002).

37. M. Topf, P. Várnai, W. G. Richards, Ab initio QM/MM dynamics simulation of the tetrahedral intermediate of serine proteases: Insights into the active site hydrogen-bonding network. *J. Am. Chem. Soc.* **124**, 14780–14788 (2002).

38. H. J. Kulik, J. Zhang, J. P. Klinman, T. J. Martinez, How large should the QM region be in QM/MM calculations? The case of catechol O-methyltransferase. *J. Phys. Chem. B.* **120**, 11381–11394 (2016).

39. T. Burgin, B. C. Pollard, B. C. Knott, H. B. Mayes, M. F. Crowley, J. E. McGeehan, G. T. Beckham, H. L. Woodcock, The reaction mechanism of the *Ideonella sakaiensis* PETase enzyme. *Commun. Chem.* **7**, 65 (2024).

40. S. Boneta, K. Arafet, V. Moliner, QM/MM study of the enzymatic biodegradation mechanism of polyethylene terephthalate. *J. Chem. Inf. Model.* **61**, 3041–3051 (2021).

41. S. Sulaiman, D.-J. You, E. Kanaya, Y. Koga, S. Kanaya, Crystal structure and thermodynamic and kinetic stability of metagenome-derived LC-cutinase. *Biochemistry* **53**, 1858–1869 (2014).

42. A. M. Baptista, P. J. Martel, S. B. Petersen, Simulation of protein conformational freedom as a function of pH: Constant-pH molecular dynamics using implicit titration. *Proteins* **27**, 523–544 (1997).

43. J. Mongan, D. A. Case, J. A. McCammon, Constant pH molecular dynamics in generalized Born implicit solvent. *J. Comput. Chem.* **25**, 2038–2048 (2004).

44. X. Kong, C. L. Brooks III, λ-dynamics: A new approach to free energy calculations. *J. Chem. Phys.* **105**, 2414–2423 (1996).

45. H. Webb, B. M. Tynan-Connolly, G. M. Lee, D. Farrell, F. O'Meara, C. R. Søndergaard, K. Teilum, C. Hewage, L. P. McIntosh, J. E. Nielsen, Remeasuring HEWL pK_a values by NMR spectroscopy: Methods, analysis, accuracy, and implications for theoretical pK_a calculations. *Proteins* **79**, 685–702 (2011).

46. N. Aho, P. Buslaev, A. Jansen, P. Bauer, G. Groenhof, B. Hess, Scalable constant pH molecular dynamics in GROMACS. *J. Chem. Theory Comput.* **18**, 6148–6160 (2022).

47. X. Fu, Z. Wu, W. Wang, T. Xie, S. Keten, R. Gomez-Bombarelli, T. Jaakkola, Forces are not enough: Benchmark and critical evaluation for machine learning force fields with molecular simulations. *Transactions on Machine Learning Research* (2023), Survey Certification, https://openreview.net/forum?id=A8pqQipwkt.

48. O. A. von Lilienfeld, K.-R. Müller, A. Tkatchenko, Exploring chemical compound space with quantum-based machine learning. *Nat. Rev. Chem.* **4**, 347–358 (2020).

49. J. S. Smith, B. Nebgen, N. Lubbers, O. Isayev, A. E. Roitberg, Less is more: Sampling chemical space with active learning. *J. Chem. Phys.* **148**, 241733 (2018).

50. J. S. Smith, O. Isayev, A. E. Roitberg, ANI-1, A data set of 20 million calculated off-equilibrium conformations for organic molecules. *Sci. Data* **4**, 170193 (2017).

51. A. P. Bartók, R. Kondor, G. Csányi, On representing chemical environments. *Phys. Rev. B* **87**, 184115 (2013).

52. J. Gilmer, S. S. Schoenholz, P. F. Riley, O. Vinyals, G. E. Dahl, "Neural message passing for quantum chemistry," in *International Conference on Machine Learning* (PMLR, 2017), pp. 1263–1272.

53. O. T. Unke, S. Chmiela, M. Gastegger, K. T. Schütt, H. E. Sauceda, K.-R. Müller, SpookyNet: Learning force fields with electronic degrees of freedom and nonlocal effects. *Nat. Commun.* **12**, 7273 (2021).

54. S. Elfwing, E. Uchibe, K. Doya, Sigmoid-weighted linear units for neural network function approximation in reinforcement learning. *Neural Netw.* **107**, 3–11 (2018).

55. J. F. Ziegler, "Stopping and range of ions in solids," in *Inelastic Near-Surface Interactions. Proceedings of the Werner Brandt Workshop*, CONF-8404190 (International Atomic Energy Agency, 1986), pp. 284–284.

56. E. Caldeweyher, S. Ehlert, A. Hansen, H. Neugebauer, S. Spicher, C. Bannwarth, S. Grimme, A generally applicable atomic-charge dependent London dispersion correction. *J. Chem. Phys.* **150**, 154122 (2019).

57. W. Falcon, The PyTorch Lightning team, PyTorch Lightning (2019); doi:10.5281/zenodo.3828935, https://github.com/Lightning-AI/lightning.

58. S. J. Reddi, S. Kale, S. Kumar, On the convergence of adam and beyond. arXiv:1904.09237 (2019).

59. P. Izmailov, D. Podoprikhin, T. Garipov, D. Vetrov, A. G. Wilson, Averaging weights leads to wider optima and better generalization. arXiv:1803.05407 (2018).

60. R. Mohammed, J. Rawashdeh, M. Abdullah, "Machine learning with oversampling and undersampling techniques: Overview study and experimental results," in *2020 11th International Conference on Information and Communication Systems (ICICS)* (IEEE, 2020), pp. 243–248.

61. S. Ahmadi, L. Barrios Herrera, M. Chehelamirani, J. Hostaš, S. Jalife, D. R. Salahub, Multiscale modeling of enzymes: QM-cluster, QM/MM, and QM/MM/MD: A tutorial review. *Int. J. Quantum Chem.* **118**, e25558 (2018).

62. E. L. Camm, G. N. Towers, Phenylalanine ammonia lyase. *Phytochemistry* **12**, 961–973 (1973).

63. O. Trott, A. J. Olson, AutoDock Vina: Improving the speed and accuracy of docking with a new scoring function, efficient optimization, and multithreading. *J. Comput. Chem.* **31**, 455–461 (2010).

64. H.-C. Chan, Y. Zhu, Y. Hu, T.-P. Ko, C.-H. Huang, F. Ren, C.-C. Chen, Y. Ma, R.-T. Guo, Y. Sun, Crystal structures of D-psicose 3-epimerase from Clostridium cellulolyticum H10 and its complex with ketohexose sugars. *Protein Cell* **3**, 123–131 (2012).

65. E. E. Ferrandi, C. Sayer, M. N. Isupov, C. Annovazzi, C. Marchesi, G. Iacobone, X. Peng, E. Bonch-Osmolovskaya, R. Wohlgemuth, J. A. Littlechild, D. Monti, Discovery and characterization of thermophilic limonene-1, 2-epoxide hydrolases from hot spring metagenomic libraries. *FEBS J.* **282**, 2879–2894 (2015).

66. M. J. Frisch, G. W. Trucks, H. B. Schlegel, G. E. Scuseria, M. A. Robb, J. R. Cheeseman, G. Scalmani, V. Barone, G. A. Petersson, H. Nakatsuji, X. Li, M. Caricato, A. V. Marenich, J. Bloino, B. G. Janesko, R. Gomperts, B. Mennucci, H. P. Hratchian, J. V. Ortiz, A. F. Iz maylov, J. L. Sonnenberg, D. Williams-Young, F. Ding, F. Lipparini, F. Egidi, J. Goings, B. Peng, A. Petrone, T. Henderson, D. Ranasinghe, V. G. Zakrzewski, J. Gao, N. Rega, G. Zheng, W. Liang,

M. Hada, M. Ehara, K. Toyota, R. Fukuda, J. Hasegawa, M. Ishida, T. Nakajima, Y. Honda, O. Kitao, H. Nakai, T. Vreven, K. Throssell, J. A. Montgomery, Jr., J. E. Peralta, F. Ogliaro, M. J. Bearpark, J. J. Heyd, E. N. Brothers, K. N. Kudin, V. N. Staroverov, T. A. Keith, R. Kobayashi, J. Normand, K. Raghavachari, A. P. Rendell, J. C. Bu rant, S. S. Iyengar, J. Tomasi, M. Cossi, J. M. Millam, M. Klene, C. Adamo, R. Cammi, J. W. Ochterski, R. L. Martin, K. Morokuma, O. Farkas, J. B. Foresman, D. J. Fox, Gaussian 16, Revision C.01 (Gaussian Inc., 2016).

67. M. Bursch, J.-M. Mewes, A. Hansen, S. Grimme, Best-practice DFT protocols for basic molecular computational chemistry. *Angew. Chem. Int. Ed. Engl.* **61**, e202205735 (2022).

68. R. M. Parrish, L. A. Burns, D. G. Smith, A. C. Simmonett, A. E. DePrince III, E. G. Hohenstein, U. Bozkaya, A. Y. Sokolov, R. Di Remigio, R. M. Richard, J. F. Gonthier, A. M. James, H. R. McAlexander, A. Kumar, M. Saitow, X. Wang, B. P. Pritchard, P. Verma, H. F. Schaefer III, K. Patkowski, R. A. King, E. F. Valeev, F. A. Evangelista, J. M. Turney, T. D. Crawford, C. D. Sherrill, Psi4 1.1: An open-source electronic structure program emphasizing automation, advanced libraries, and interoperability. *J. Chem. Theory Comput.* **13**, 3185–3197 (2017).

69. C. Adamo, V. Barone, Toward reliable density functional methods without adjustable parameters: The PBE0 model. *J. Chem. Phys.* **110**, 6158–6170 (1999).

70. A. Tkatchenko, R. A. DiStasio Jr, R. Car, M. Scheffler, Accurate and efficient method for many-body van der Waals interactions. *Phys. Rev. Lett.* **108**, 236402 (2012).

71. K. Schütt, P. Kessel, M. Gastegger, K. A. Nicoli, A. Tkatchenko, K.-R. Müller, SchNetPack: A deep learning toolbox for atomistic systems. *J. Chem. Theory Comput.* **15**, 448–455 (2018).

72. H. J. Berendsen, J. P. Postma, W. F. van Gunsteren, J. Hermans, “Interaction models for water in relation to protein hydration,” in *Intermolecular Forces: Proceedings of the Fourteenth Jerusalem Symposium on Quantum Chemistry and Biochemistry* (Springer, 1981), pp. 331–342.

73. E. Helfand, Brownian dynamics study of transitions in a polymer chain of bistable oscillators. *J. Chem. Phys.* **69**, 1010–1018 (1978).

74. M. Meuwly, M. Karplus, Simulation of proton transfer along ammonia wires: An “ab initio” and semiempirical density functional comparison of potentials and classical molecular dynamics. *J. Chem. Phys.* **116**, 2572–2585 (2002).

75. W. Zeng, X. Li, Y. Yang, J. Min, J.-W. Huang, W. Liu, D. Niu, X. Yang, X. Han, L. Zhang, L. Dai, C. C. Chen, R. T. Guo, Substrate-binding mode of a thermophilic PET hydrolase and engineering the enzyme to enhance the hydrolytic efficacy. *ACS Catal.* **12**, 3033–3040 (2022).

76. N. M. O'Boyle, M. Banck, C. A. James, C. Morley, T. Vandermeersch, G. R. Hutchison, Open Babel: An open chemical toolbox. *J. Chem.* **3**, 33 (2011).

77. D. A. Case, H. M. Aktulga, K. Belfon, D. S. Cerutti, G. A. Cisneros, V. W. D. Cruzeiro, N. Forouzesh, T. J. Giese, A. W. Gotz, H. Gohlke, S. Izadi, K. Kasavajhala, M. C. Kaymak, E. King, T. Kurtzman, T.-S. Lee, P. Li, J. Liu, T. Luchko, R. Luo, M. Manathunga, M. R. Machado, H. M. Nguyen, K. A. O’Hearn, A. V. Onufriev, F. Pan, S. Pantano, R. Qi, A. Rahnamoun, A. Risheh, S. Schott-Verdugo, A. Shajan, J. Swails, J. Wang, H. Wei, X. Wu, Y. Wu, S. Zhang, S. Zhao, Q. Zhu, T. E. Cheatham III, D. R. Roe, A. Roitberg, C. Simmerling, D. M. York, M. C. Nagan, K. M. Merz Jr, AmberTools. *J. Chem. Inf. Model.* **63**, 6183–6191 (2023).

78. D. C. Liu, J. Nocedal, On the limited memory BFGS method for large scale optimization. *Math. Program.* **45**, 503–528 (1989).

79. D. P. Kovács, J. H. Moore, N. J. Browning, I. Batatia, J. T. Horton, Y. Pu, V. Kapil, W. C. Witt, I.-B. Magdau, D. J. Cole, G. Csányi, Mace-off: Short-range transferable machine learning force fields for organic molecules. *J. Am. Chem. Soc.* **147**, 17598–17611 (2025).

80. D. M. Anstine, R. Zubatyuk, O. Isayev, AIMNet2: A neural network potential to meet your neutral, charged, organic, and elemental-organic needs. *Chem. Sci.* **16**, 10228–10244 (2025).
